# Computerized Clinical Decision Support Systems for the Early Detection of Sepsis Among Adult Inpatients: Scoping Review

**DOI:** 10.2196/31083

**Published:** 2022-02-23

**Authors:** Khalia Ackermann, Jannah Baker, Malcolm Green, Mary Fullick, Hilal Varinli, Johanna Westbrook, Ling Li

**Affiliations:** 1 Centre for Health Systems and Safety Research Australian Institute of Health Innovation Macquarie University Australia; 2 Clinical Excellence Commission Sydney Australia; 3 eHealth New South Wales Sydney Australia

**Keywords:** sepsis, early detection of disease, clinical decision support systems, patient safety, electronic health records, sepsis care pathway

## Abstract

**Background:**

Sepsis is a significant cause of morbidity and mortality worldwide. Early detection of sepsis followed promptly by treatment initiation improves patient outcomes and saves lives. Hospitals are increasingly using computerized clinical decision support (CCDS) systems for the rapid identification of adult patients with sepsis.

**Objective:**

This scoping review aims to systematically describe studies reporting on the use and evaluation of CCDS systems for the early detection of adult inpatients with sepsis.

**Methods:**

The protocol for this scoping review was previously published. A total of 10 electronic databases (MEDLINE, Embase, CINAHL, the Cochrane database, LILACS [Latin American and Caribbean Health Sciences Literature], Scopus, Web of Science, OpenGrey, ClinicalTrials.gov, and PQDT [ProQuest Dissertations and Theses]) were comprehensively searched using terms for sepsis, CCDS, and detection to identify relevant studies. Title, abstract, and full-text screening were performed by 2 independent reviewers using predefined eligibility criteria. Data charting was performed by 1 reviewer with a second reviewer checking a random sample of studies. Any disagreements were discussed with input from a third reviewer. In this review, we present the results for adult inpatients, including studies that do not specify patient age.

**Results:**

A search of the electronic databases retrieved 12,139 studies following duplicate removal. We identified 124 studies for inclusion after title, abstract, full-text screening, and hand searching were complete. Nearly all studies (121/124, 97.6%) were published after 2009. Half of the studies were journal articles (65/124, 52.4%), and the remainder were conference abstracts (54/124, 43.5%) and theses (5/124, 4%). Most studies used a single cohort (54/124, 43.5%) or before-after (42/124, 33.9%) approach. Across all 124 included studies, patient outcomes were the most frequently reported outcomes (107/124, 86.3%), followed by sepsis treatment and management (75/124, 60.5%), CCDS usability (14/124, 11.3%), and cost outcomes (9/124, 7.3%). For sepsis identification, the systemic inflammatory response syndrome criteria were the most commonly used, alone (50/124, 40.3%), combined with organ dysfunction (28/124, 22.6%), or combined with other criteria (23/124, 18.5%). Over half of the CCDS systems (68/124, 54.8%) were implemented alongside other sepsis-related interventions.

**Conclusions:**

The current body of literature investigating the implementation of CCDS systems for the early detection of adult inpatients with sepsis is extremely diverse. There is substantial variability in study design, CCDS criteria and characteristics, and outcomes measured across the identified literature. Future research on CCDS system usability, cost, and impact on sepsis morbidity is needed.

**International Registered Report Identifier (IRRID):**

RR2-10.2196/24899

## Introduction

### Sepsis and Early Detection

Sepsis, defined in 2016 as “life-threatening organ dysfunction caused by a dysregulated host response to infection,” is a leading cause of death worldwide [1]. A recent study by Rudd et al [2] estimated that 48.9 million cases of sepsis were reported in 2017, with 11 million sepsis-related deaths, representing 1 in 5 of all deaths globally [2]. Furthermore, survivors of sepsis often have a decreased quality of life, including higher rates of mortality, physical disabilities, chronic illnesses, mental health issues, and cognitive impairments [3-9].

Prompt administration of sepsis therapies, such as intravenous antimicrobials and fluid resuscitation, is associated with better patient outcomes and lower health care–related costs [10,11]. Therefore, it is critical to detect sepsis as early as possible to ensure rapid initiation of treatment [12-14]. Unfortunately, sepsis has no diagnostic gold standard and extremely heterogenous signs and symptoms, making it difficult for clinicians to distinguish it from other acute conditions [15]. The use of sepsis identification tools, such as the Quick Sepsis-Related Organ Failure Assessment, the National Early Warning Score, and the Adult Sepsis Pathway, helps facilitate early sepsis recognition [16-18]. However, these tools typically rely on manual input of vital sign information and score calculation by clinicians. Thus, timely sepsis identification hinges on vigilant and regular patient monitoring [19]. These difficulties often result in delayed sepsis diagnosis and treatment in hospitals [19,20].

### Computerized Clinical Decision Support Systems

The extensive implementation of data-rich electronic health records in health institutions has brought the opportunity for widespread integration of digital health care support systems [21]. In particular, the incorporation of computerized clinical decision support (CCDS) into hospital systems has the potential to assist accurate and timely early sepsis detection. CCDS systems can be designed with integrated sepsis-risk warning tools that alert clinicians to patients at risk of sepsis [13,22], reducing the physical and mental workload associated with manual patient monitoring [21].

Over the past 10 years, CCDS technology has rapidly expanded, with two distinct approaches emerging: knowledge-based CCDS systems programmed with predefined rules derived from established clinical knowledge and adaptive CCDS systems using artificial intelligence and machine learning techniques [21,23,24]. In this scoping review, we focused on the use of knowledge-based CCDS systems in sepsis detection.

### Research Questions and Aims

The use and implementation of sepsis CCDS systems in real-world clinical settings is a novel, rapidly expanding, and highly complex field [21,25]. In this scoping review, we systematically mapped the literature available on sepsis CCDS systems with the intention of identifying knowledge gaps and informing future research. The research question directing this review is *What is the evidence base for the use of knowledge-based clinical decision support systems in hospitals for early sepsis detection and how have they been evaluated?*

More specifically, through this scoping review, we aim to (1) scope the study contexts, designs, and research methods used; (2) summarize the study outcomes investigated; and (3) map the range of CCDS system designs and implementation features, such as sepsis clinical criteria.

## Methods

### Overview

The detailed methodology for conducting this scoping review was published previously in a protocol [26]. In brief, the review was guided by the Joanna Briggs Institute Reviewer’s Manual [27], the PRISMA-ScR (Preferred Reporting Items for Systematic Reviews and Meta-Analyses extension for Scoping Reviews) [28], and the 5-stage scoping review framework proposed by Arksey and O’Malley [29]. A search for current reviews and protocols on this topic was undertaken and confirmed the absence of scoping reviews. A completed PRISMA-ScR checklist is attached in Multimedia Appendix 1 [28].

### Study Selection

We used a broad 3-step search strategy, as outlined in our protocol [26]. An experienced librarian was consulted to help construct and refine the search. The final search strategy combined terms relating to sepsis with CCDS and detection, while excluding artificial intelligence, and was used to search MEDLINE, Embase, CINAHL, the Cochrane database, LILACS (Latin American and Caribbean Health Sciences Literature), Scopus, Web of Science, OpenGrey, ClinicalTrials.gov, and PQDT (ProQuest Dissertations and Theses Global). We restricted the search to human studies in the English language. An example of the final strategy adapted for MEDLINE can be seen in Multimedia Appendix 2. The database search was undertaken in September 2020, with no date limits applied. The reference lists of relevant systematic reviews were hand-searched to identify additional studies. Any studies identified via hand searching up until the end of data extraction (early 2021) were included. We included both peer-reviewed journal articles and gray literature (ie, conference abstracts and theses).

**Figure 1:**
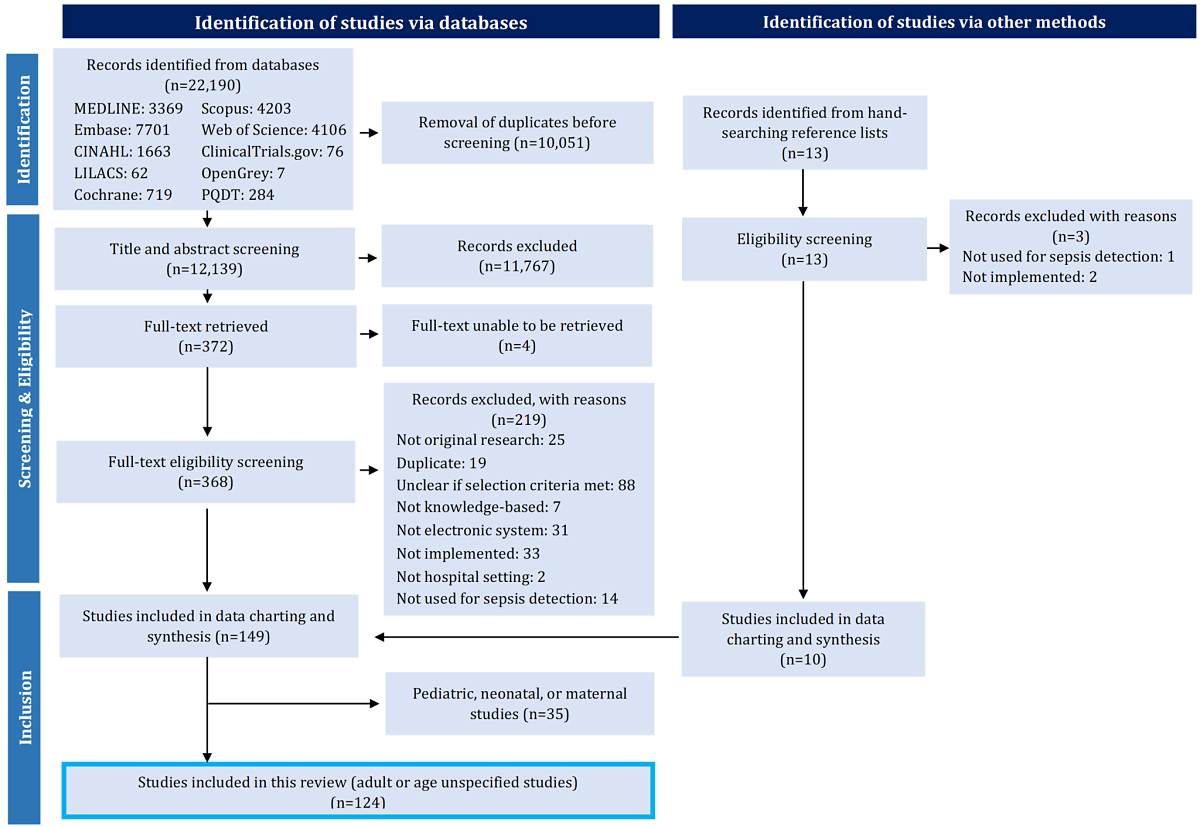
PRISMA (Preferred Reporting Items for Systematic Reviews and Meta-Analyses) flowchart demonstrating the study selection process. LILACS: Latin American and Caribbean Health Science Literature; PQDT: ProQuest Dissertations and Theses Global.

Following the search, duplicates were removed as was gray literature that had been published as a peer-reviewed journal article. However, we kept studies if they reported the same methods and study cohort but examined different outcomes. Using the eligibility criteria as reported in our protocol [26], 2 reviewers (KA and JB) independently performed title, abstract, and full-text screening, with any disagreements resolved through discussion or review by a third researcher (LL). Title and abstract screening was piloted with a random selection of 25 studies by both reviewers (KA and JB). Similarly, full-text screening was piloted with a random selection of 10 studies. The 2 reviewers (KA and JB) had 100% agreement during the title and abstract screen pilot, 97.6% agreement for the full title and abstract screen, 60% agreement for the full-text screen pilot, and 77.4% agreement for the full-text screen. Hand searching was completed by 1 reviewer (KA) with identified studies confirmed by a second (JB). A PRISMA (Preferred Reporting Items for Systematic Reviews and Meta-Analyses) flow diagram visually illustrating this process is shown in Figure 1.

Following screening, it was determined that the results of this review would be split over 2 papers, one investigating adult or unspecified populations and another investigating pediatric, neonatal, and maternal populations. Pediatric, neonatal, and maternal populations have remarkably different sepsis presentations and physiology compared with the general adult population [30-32]. The separation of results will allow for a more meaningful analysis. Included studies with unspecified age were assumed to likely include all patients in a general hospital setting and were grouped with adult populations. This paper reports the results of all studies investigating CCDS systems studied in adults or populations with an unspecified age.

### Data Abstraction

The data charting form used was iteratively designed based on the study aims. The form was piloted by a single reviewer (KA) and double-checked by a second (JB). Changes to the form were made following discussion between 3 reviewers (KA, JB, and LL). Data charting was performed by 1 reviewer (KA) with ongoing consultation with the review team.

The final data charting form included the components listed in our protocol [26], with minor adjustments as reported in Multimedia Appendix 3 [33-39]. Notably, an additional category *clarity of outcome reporting* was added to the form to account for the variability in outcome reporting transparency. Studies were categorized as having *good* clarity of outcome reporting if they specified the primary outcomes, the outcome analysis method, and the outcome measure definitions and *poor* clarity if the outcomes were not clearly described or there was a substantial reporting discrepancy between the methods and the results. Studies were categorized as having *average* clarity if they fulfilled some criteria of both good and poor.

We accepted any definition of charted data items as specified by the studies. For example, we accepted any definition of systemic inflammatory response syndrome (SIRS), any definition of sepsis, or any cost outcomes specified for the CCDS system. We defined the usability outcome category to follow the ISO definition of usability from ISO 9241-11:2018, section 3.1.1: “extent to which a system, product or service can be used by specified users to achieve specified goals with effectiveness, efficiency, and satisfaction in a specific context of use” [33]. We required usability outcomes to be specifically investigated from the perspective of end point users (ie, clinicians). To match this definition change, usability outcomes were retrospectively categorized into the effectiveness, efficiency, or satisfaction of the CCDS system from the user’s perspective.

### Analyzing and Reporting the Results

The results were analyzed through both a narrative review and quantitative descriptive analysis. A narrative summary of the data is presented, organized by our 3 aims. The data charted for each aim are summarized into tables using frequency counts and percentages. Graphical figures were also produced, where appropriate.

Owing to the extensive scope of the data charted, several subgroups were collapsed into larger groups to avoid issues of small cell size and to allow for a more meaningful summary. A complete list of the smaller subgroups condensed into the larger groups, organized by table and figure, can be found in Multimedia Appendix 4. Of note, nurses were frequently reported as CCDS system responding personnel and so were grouped separately from other clinicians to better highlight this.

### Ethics

Ethical approval or consent to participate was not required for the scoping review. The data were charted from published studies, and no individual information was included.

## Results

### Study Characteristics

Our initial search identified 22,190 studies, with 12,139 remaining after duplicate removal. Following title, abstract, and full-text screening, 149 studies met our inclusion criteria (Figure 1). Hand searching identified 10 additional studies, resulting in a total of 159 included studies. Of these 159 studies, 124 investigated adult or unspecified populations and were included in this manuscript (Figure 1). A table detailing the main study characteristics for all 124 included studies can be found in Multimedia Appendix 5 [40-163]. In total, 52.4% (65/124) of the studies were categorized as journal articles, 43.5% (54/124) as conference abstracts, and 4% (5/124) as theses (Multimedia Appendices 4 and 5).

### Aim 1: Study Context and Design

The context and design characteristics of the studies included in this review are presented in Table 1. Of the 124 included studies, 111 (89.5%) used purely quantitative methods to evaluate CCDS systems (Table 1). Most studies (96/124, 77.4%) used either single cohort (54/124, 43.5%) or before-after (42/124, 33.9%) study designs (Table 1). Very few studies used more robust study designs, such as randomized controlled trials (5/124, 4%), controlled studies (7/124, 5.6%), or interrupted time series (4/124, 3.2%; Table 1). None of the studies reported the use of reporting guidelines. An approximately even distribution of studies was observed across different hospital settings, such as hospital-wide, and specific settings (eg, intensive care unit [ICU], emergency department, and inpatient wards; Table 1).

**Figure 2:**
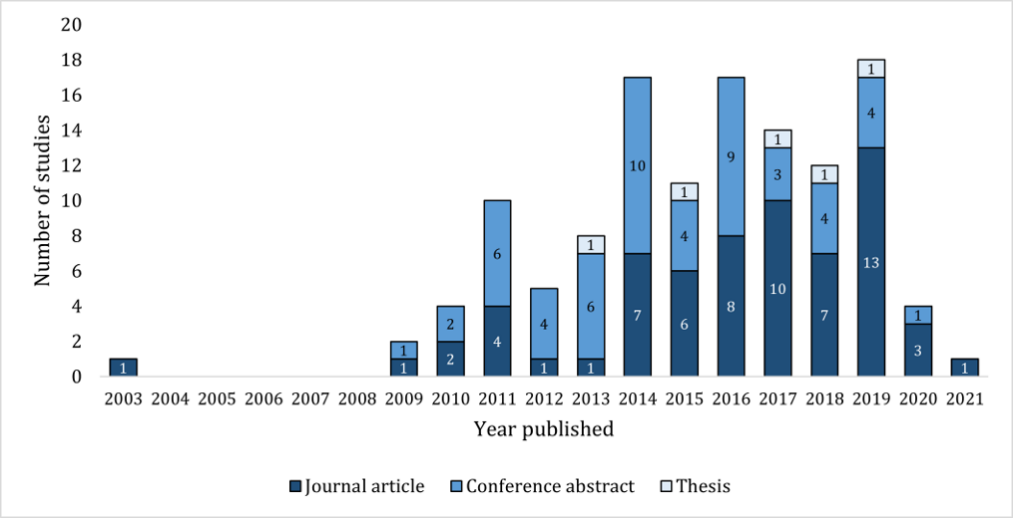
Number of studies by publication type and year published. Studies published in 2020 include those until September 2020. Studies published in 2021 were found through hand searching.

All studies but 1 (123/124, 99.2%) were published from 2009 onwards, and of the journal articles, 85% (55/65) were published in 2014 or later (Figure 2). Overall, the number of journal articles published steadily increased over time. Of the 65 journal articles, 46 (71%) reported studies conducted in the United States; 2 (3%) each in Germany, Saudi Arabia, and the United Kingdom; 1 (2%) each in Australia, Brazil, Israel, and South Korea; and 9 (14%) did not report which country they were conducted in (Multimedia Appendix 6).

Just over half (66/124, 53.2%) of the studies specified the age of the population as adult. Within these studies, there was a reasonable variation in the actual age range provided. Almost half (29/66, 44%) reported an adult population aged ≥18 years, whereas 30% (20/66) of the studies did not specify an age range further than *adult*. The remaining studies reported populations using thresholds such as aged >14 (1/66, 2%), ≥14 (3/66, 5%), >16 (2/66, 3%), ≥16 (3/66, 5%), ≥19 (6/66, 9%), and ≥70 (1/66, 2%) years, with 2% (1/66) of the studies inconsistently listing multiple thresholds.

**Table 1:** Study context and design.

Study characteristics			Studies, n (%)							Total (N=124), n (%)
			Conference abstract (n=54)		Journal article (n=65)		Thesis (n=5)			
**Method**										
	Quantitative	50 (92.6)		56 (86.2)		5 (100)		111 (89.5)		
	Qualitative	1 (1.9)		5 (7.7)		0 (0)		6 (4.8)		
	Mixed methods	3 (5.6)		4 (6.2)		0 (0)		7 (5.6)		
**Principal study type**										
	Surveys or focus groups or heuristics	1 (1.9)		5 (7.7)		0 (0)		6 (4.8)		
	Case control	1 (1.9)		0 (0)		0 (0)		1 (0.8)		
	Single cohort	30 (55.6)		22 (33.8)		2 (40)		54 (43.5)		
	Before and after	13 (24.1)		27 (41.5)		2 (40)		42 (33.9)		
	Interrupted time series	0 (0)		3 (4.6)		1 (20)		4 (3.2)		
	Controlled study	3 (5.6)		4 (6.2)		0 (0)		7 (5.6)		
	Randomized controlled trial	2 (3.7)		3 (4.6)		0 (0)		5 (4)		
	Insufficient information to determine	4 (7.4)		1 (1.5)		0 (0)		5 (4)		
**Setting**										
	Hospital-wide^a^	9 (16.7)		17 (26.2)		1 (20)		27 (21.8)		
	Intensive care unit	11 (20.4)		14 (21.5)		1 (20)		26 (21)		
	Emergency department	18 (33.3)		16 (24.6)		2 (40)		36 (29)		
	Inpatient wards	11 (20.4)		12 (18.5)		1 (20)		24 (19.4)		
	Specific ward	5 (9.3)		6 (9.2)		0 (0)		11 (8.9)		
**Number of sites**										
	1	32 (59.3)		37 (56.9)		2 (40)		71 (57.3)		
	2-5	6 (11.1)		16 (24.6)		3 (60)		25 (20.2)		
	>5	0 (0)		6 (9.2)		0 (0)		6 (4.8)		
	Unspecified	16 (29.6)		6 (9.2)		0 (0)		22 (17.7)		
**Age group specified?**										
	Yes	18 (33.3)		44 (67.7)		4 (80)		66 (53.2)		
	No	36 (66.7)		21 (32.3)		1 (20)		58 (46.8)		
**Number of participants**										
	<100	3 (5.6)		8 (12.3)		0 (0)		11 (8.9)		
	101-500	11 (20.4)		14 (21.5)		1 (20)		26 (21)		
	501-1000	6 (11.1)		4 (6.2)		0 (0)		10 (8.1)		
	1001-10,000	9 (16.7)		12 (18.5)		1 (20)		22 (17.7)		
	>10,001	10 (18.5)		15(23.1)		2 (40)		27 (21.8)		
	Unspecified	15 (27.8)		12 (18.5)		1 (20)		28 (22.6)		
**Funding**										
	Yes	3 (5.6)		26 (40)		1 (20)		30 (24.2)		
	No	2 (3.7)		13 (20)		0 (0)		15 (12.1)		
	Unspecified	49 (90.7)		26 (40)		4 (80)		79 (63.7)		

^a^If the study setting was not explicitly stated, it was assumed to be hospital-wide.

### Aim 2: Study Outcomes

**Figure 3:**
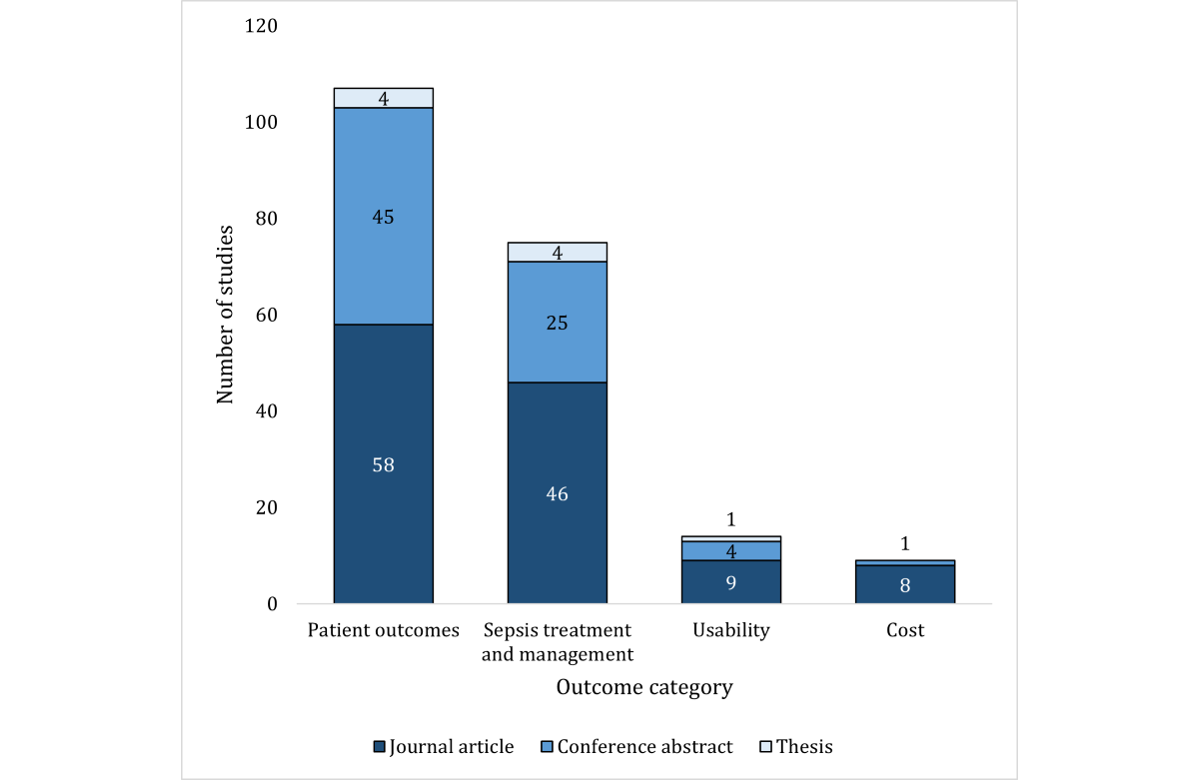
Proportion of studies reporting each outcome category.

The outcomes investigated by the included journal articles and the conference abstracts and theses are presented in Table 2 and Multimedia Appendix 7, respectively. Of the 4 predefined outcome categories, patient outcomes were reported in the highest number of studies (107/124, 86.3%; Figure 3). Sepsis treatment and management outcomes were reported in 60.5% (75/124) of the studies, CCDS system usability outcomes in 11.3% (14/124), and cost outcomes in 7.3% (9/124; Figure 3).

**Figure 4:**
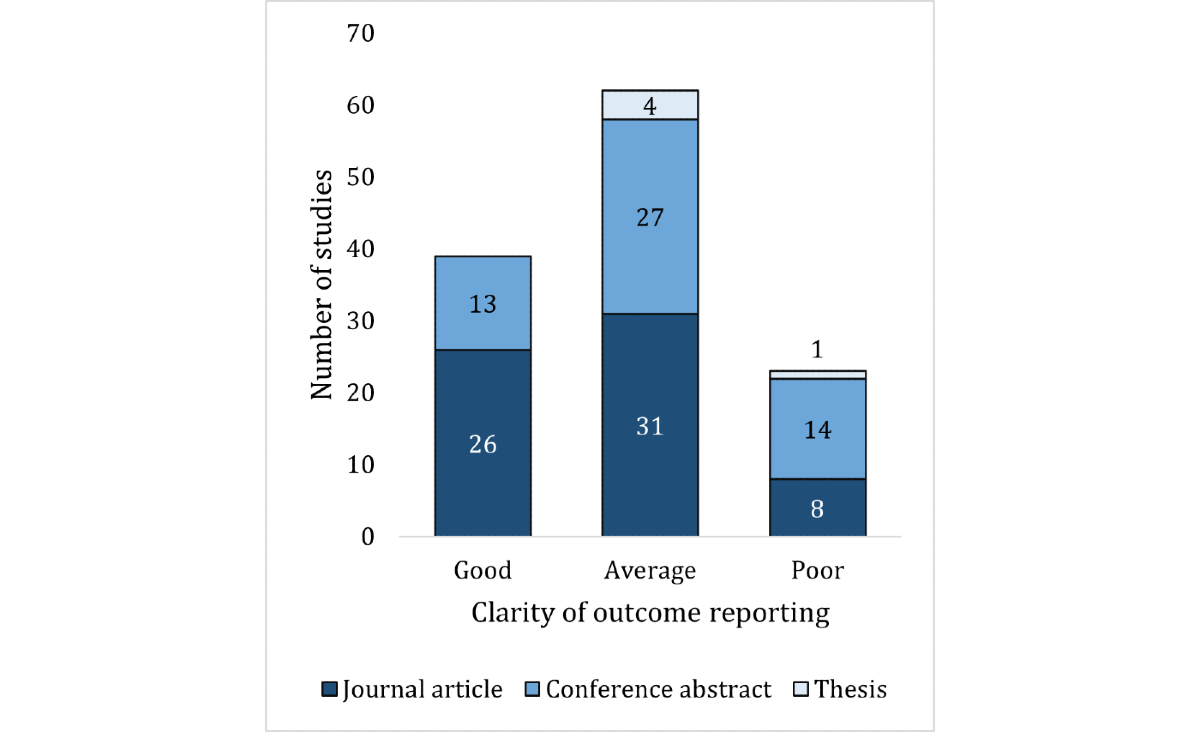
Clarity of outcome reporting in the studies.

Overall, only 31.5% (39/124) of the studies had good clarity in outcome reporting (Figure 4). Generally, studies had average (62/124, 50%) or poor clarity (23/124, 18.5%). Unsurprisingly, journal articles had better clarity of outcome reporting, with 40% (26/65) of the articles having good clarity, compared with 22% (13/59) of the conference abstracts or theses (Figure 4).

In the 65 journal articles, mortality was the most frequently reported patient outcome (39/65, 60%). Overall, 35 different types of mortality measures were reported 55 times across 39 studies (Multimedia Appendix 8). Of these, in-hospital mortality was the most frequently reported (13/55, 24%; Multimedia Appendix 8). Sepsis identification, length of stay, and *other* patient outcomes were also frequently reported, appearing in 38% (25/65), 34% (22/65), and 35% (23/65) of the articles, respectively (Table 2; see Multimedia Appendix 4 for the expanded list of included outcomes). ICU admission was the least reported patient outcome (12/65, 18%). In the sepsis treatment and management outcome category, antibiotic-related and *other* were the most frequently reported outcomes in journal articles (27/65, 42% and 31/65, 48%, respectively), followed by lactate-, fluids-, and blood culture–related outcomes (17/65, 26%; 14/65, 22%; and 14/65, 22%, respectively; Table 2; see Multimedia Appendix 4 for expanded list of included outcomes). Overall, sepsis bundle or protocol compliance was the least reported outcome in journal articles (12/65, 18%).

CCDS system usability outcomes were reported in similar numbers of journal articles, with 12% (8/65) of the journal articles reporting on the efficiency of the system, 11% (7/65) on system effectiveness, and 11% (7/65) reporting on users’ satisfaction with the system (Table 2). Among the CCDS system-related cost outcomes, cost was reported in the greatest number of journal articles (5/65, 8%), whereas cost-effectiveness or savings were reported in only 5% (3/65) of the articles (Table 2).

**Table 2:** Main outcomes and outcome categories in journal articles.

Outcome categories		Outcome classification^a^, n (%^b^)				Total, (n=65), n (%)^c^
		Primary	Secondary	Not specified^d^		
**Patient outcomes**						
	Mortality	11 (28)	9 (23)	19 (49)	39 (60)	
	Sepsis identification	10 (40)	3 (12)	12 (48)	25 (38)	
	Length of stay	3 (14)	10 (45)	9 (41)	22 (34)	
	Intensive care unit admission	0 (0)	5 (42)	7 (58)	12 (18)	
	Other	4 (17)	8 (35)	11 (48)	23 (35)	
**Sepsis treatment and management**						
	Antibiotics	6 (22)	9 (33)	12 (44)	27 (42)	
	Lactate	2 (12)	6 (35)	9 (53)	17 (26)	
	Fluids	2 (14)	4 (29)	8 (57)	14 (22)	
	Blood culture	2 (14)	4 (29)	8 (57)	14 (22)	
	Sepsis bundle or protocol compliance	2 (17)	4 (33)	6 (50)	12 (18)	
	Other	5 (16)	7 (23)	19 (61)	31 (48)	
**Usability**						
	Efficiency	0 (0)	2 (25)	6 (75)	8 (12)	
	Effectiveness	0 (0)	1 (14)	6 (86)	7 (11)	
	Satisfaction	0 (0)	3 (43)	4 (57)	7 (11)	
**Cost**						
	Cost	1 (20)	2 (40)	2 (40)	5 (8)	
	Cost-effectiveness or savings	0 (0)	1 (33)	2 (67)	3 (5)	

^a^Some studies reported both primary, secondary, or nonspecified outcomes within the same outcome group. To avoid double-counting these studies, secondary outcomes were not counted in favor of counting primary outcomes. Similarly, nonspecified outcomes were not counted in favor of primary or secondary outcomes. For example, a study may have the primary outcome mortality (30-day) and the secondary outcome mortality (7-day), which would both fall into the mortality outcome group. In this example the study would be counted as having mortality as the primary outcome.

^b^These percentages were calculated as row percentages, that is, using the number in the “Total” column in each row as the denominator.

^c^The percentages were calculated from the number of journal articles (n=65), not the number of total outcomes. As many journal articles reported multiple outcomes, there were more than 65 outcomes in each category, and therefore, the percentages will add up to more than 100%.

^d^The study did not specify whether the outcome was primary or secondary.

### Aim 3: CCDS Characteristics

The characteristics of the CCDS systems reported in the included studies are presented in Table 3. Half (64/124, 51.6%) of the studies, most of which were journal articles (44/64, 69%), implemented homegrown CCDS systems. Of the 124 studies, only 13 (10.5%), including 10 (77%) journal articles, implemented commercial CCDS systems, of which 69% (9/13) were the St John’s Sepsis Surveillance Agent (Cerner Corporation, Kansas City, Missouri, United States; Table 3). Most included studies (95/124, 76.6%) evaluated *live* CCDS systems only, where the CCDS was implemented and actively sending alerts. Silent CCDS, where the system would run in real time but not send clinical alerts, were implemented by 7.3% (9/124) of studies, and 11.3% (14/124) of studies implemented both silent and live CCDS, either sequentially or concurrently.

SIRS alone was the most frequently used CCDS clinical criteria for sepsis identification (50/124, 40.3%), followed by SIRS combined with organ dysfunction (28/124, 22.6%), and SIRS combined with other criteria (23/124, 18.5%; Table 3). In addition, a diverse range of other criteria were used by 32.3% (40/124) of studies (Multimedia Appendix 4), while 7.3% (9/124) did not specify the clinical criteria used (Table 3)

Over half of the studies reported the implementation of CCDS systems alongside numerous other related interventions (68/124, 54.8%), such as staff education programs and antibiotic order sets (Table 3). The most common type of concurrent intervention used was clinical protocols in 41.9% (52/124) of the studies (Table 3).

Most commonly, studies reported nurses (51/124, 41.1%) or other clinicians (37/124, 29.8%) as the main CCDS alert responding personnel (Table 3). Some studies reported on CCDS with response teams (12/124, 9.7%), study coordinators (8/124, 6.5%), or other personnel (11/124, 8.9%) responding to the alerts. Of the 124 studies, 33 (26.6%) reported the use of the electronic health record to distribute CCDS alerts, 26 (21%) the use of pagers, 15 (12.1%) the use of a patient dashboard or work list, and 8 (6.5%) the use of another form of alert delivery (Table 3).

**Table 3:** Computerized clinical decision support (CCDS) characteristics.

CCDS characteristic			Studies, n (%^a^)				Total (N=124), n (%^a^)
			Conference abstract (n=54)	Journal article (n=65)	Thesis (n=5)		
**CCDS type**							
	Homegrown		18 (33.3)	44 (67.7)	2 (40)	64 (51.6)	
	**Commercial**		3 (5.6)	10 (15.4)	0 (0)	13 (10.5)	
		St John sepsis (Cerner Corporation, Kansas City, Missouri, United States)	2 (3.7)	7 (10.7)	0 (0)	9 (7.3)	
		PREDEC ALARM (Löser Medizintechnik GmbH, Leipzig, Germany)	0 (0)	1 (1.5)	0 (0)	1 (0.8)	
		Unspecified	1 (1.9)	2 (3.1)	0 (0)	3 (2.4)	
	Unspecified		33 (61.1)	11 (16.9)	3 (60)	47 (37.9)	
**Silent or live?**							
	Live		41 (75.8)	49 (75.4)	5 (100)	95 (76.6)	
	Silent		5 (9.3)	4 (6.2)	0 (0)	9 (7.3)	
	Both		4 (7.4)	10 (15.4)	0 (0)	14 (11.3)	
	Unspecified		4 (7.4)	2 (3.1)	0 (0)	6 (4.8)	
**CCDS criteria**							
	SIRS^b^		24 (44.4)	24 (36.9)	2 (40)	50 (40.3)	
	SIRS + organ dysfunction		11 (20.4)	17 (26.2)	0 (0)	28 (22.6)	
	SIRS + other		9 (16.7)	12 (18.5)	2 (40)	23 (18.5)	
	Other		17 (31.5)	23 (35.4)	0 (0)	40 (32.3)	
	Unspecified		3 (5.6)	5 (7.7)	1 (20)	9 (7.3)	
**Related interventions**							
	Clinical protocol		16 (29.6)	34 (52.3)	2 (40)	52 (41.9)	
	Education and staff resources		8 (14.8)	25 (38.5)	1 (20)	34 (27.4)	
	Electronic or infrastructure changes		2 (3.7)	6 (9.2)	0 (0)	8 (6.5)	
	Response or leadership team		4 (7.4)	17 (26.2)	1 (20)	22 (17.7)	
	Order sets		10 (18.5)	17 (26.2)	0 (0)	27 (21.8)	
	Feedback		0 (0)	10 (15.4)	0 (0)	10 (8.1)	
	None		32 (59.3)	22 (33.8)	2 (40)	56 (45.2)	
**Responding personnel**							
	Nurses^c^		12 (22.2)	38 (58.5)	1 (20)	51 (41.1)	
	Other clinicians		11 (20.4)	26 (40)	0 (0)	37 (29.8)	
	Response team		4 (7.4)	8 (12.3)	0 (0)	12 (9.7)	
	Study coordinator		0 (0)	8 (12.3)	0 (0)	8 (6.5)	
	Other		4 (7.4)	6 (9.2)	1 (20)	11 (8.9)	
	Unspecified		27 (50)	7 (10.8)	3 (60)	37 (29.8)	
**Alert delivery**							
	Electronic patient record		6 (11.1)	27 (41.5)	0 (0)	33 (26.6)	
	Pager		4 (7.4)	20 (30.8)	2 (40)	26 (21.0)	
	Patient dashboard or working list		4 (7.4)	10 (15.4)	1 (20)	15 (12.1)	
	Other		1 (1.9)	6 (9.2)	1 (20)	8 (6.5)	
	Unspecified		42 (77.8)	19 (29.2)	2 (40)	63 (50.8)	

^a^As some studies reported multiple characteristics within each category, there were more than the total number of studies, and therefore, the percentages may add up to more than 100%.

^b^SIRS: systemic inflammatory response syndrome.

^c^As nurses are frequently reported as CCDS system responding personnel, they were grouped separately from other clinicians.

## Discussion

### Principal Findings

This review canvassed 124 studies in total, representing a comprehensive overview of current research, including an extensive body of gray literature. Over half of the included studies were journal articles (65/124, 52.4%), and nearly all studies were published in the last decade, indicating the considerable volume of recent research investigating the use of CCDS systems for early detection of adult inpatients with sepsis. Our findings demonstrate the substantial diversity of studies across all three aims: (1) the context and design of the study, (2) the type and measurement of outcomes investigated, and (3) the design and implementation of the CCDS system evaluated. We identified little research into the effects of CCDS on patient morbidity or CCDS usability and cost outcomes, highlighting key knowledge gaps in the literature. Our review also underlines the need for robust study designs, as well as improved generalizability and reporting in future studies.

### Variability Across Studies

There is extensive heterogeneity in the current literature investigating the implementation and evaluation of CCDS systems for early sepsis detection in adult hospital patients. In particular, there was considerable diversity displayed in the chosen clinical criteria for sepsis identification across the studies included in our review. Although many studies used the SIRS criteria, alone or with adjuncts, there was a substantial range of other criteria used (Multimedia Appendix 4). This can be attributed to the extremely diverse presentations of patients with sepsis, which has led to the development of numerous different clinical scores for sepsis detection [15-18,164]. In addition, our findings demonstrated variability in the method of alert delivery, personnel who respond to alerts, and concurrent implementation of related interventions. Studies were conducted across a range of different hospital settings, including hospital-wide or specific sites, such as the emergency department or ICU (Table 1). The chosen threshold for what age participants were included in the study was also quite variable, with studies defining their *adult* population using cutoff points ranging from 14 to 19 years and older. Finally, our review illuminated the expansive number of outcomes used to evaluate and investigate sepsis CCDS systems. Previous systematic reviews have similarly highlighted this diversity [13,21,22,165,166]. This heterogeneity across settings, participants, CCDS system characteristics, and outcomes makes it difficult to compare studies and to make general statements regarding sepsis CCDS systems.

This diversity can be partially attributed to the novel nature of sepsis CCDS systems and the recent emergence of the field. Our findings show a vast expansion of the literature, with three-quarters of studies published since 2014 (Figure 2). Owing to this recent rapid development of the field and the simultaneous evolution of information and communication technology in health care [21,167], there is no well-established research strategy or dogma for this specific area. Consequently, different authors have designed and executed their studies using a diverse range of variables and study design methodologies.

This variability can also be attributed to the complexity involved in the implementation of health care interventions [168,169]. To characterize this complexity, Greenhalgh et al [170] have designed the NASSS (nonadoption, abandonment, scale-up, spread, and sustainability) framework. In the case of CCDS systems for early sepsis detection in hospitals, the 7 NASSS framework domains can be identified as follows: sepsis (the condition); the CCDS system (the technology); the commercial and health-associated value of CCDS systems (value proposition and value chain); the responding personnel (the adopters); the hospital setting (the organization); the local, state, or national health system (the wider system); and software plasticity (embedding and adaptation over time). Our findings demonstrate that these domains are extremely diverse across the included studies, presenting many variables and variable combinations, consequentially expanding the complexity involved in sepsis CCDS system implementation. As the complexity of a system has been associated with its capacity for successful and sustainable implementation [25], this heterogeneity could detrimentally impact the performance of sepsis CCDS systems. To counter this issue, Greenhalgh et al [25] highlights the importance of system usability and adaptability, suggesting that a user-centered and iterative approach is needed, centralizing the involvement of relevant providers in the implementation plan. Unfortunately, our findings indicate that few of the included studies investigated the usability of sepsis CCDS systems.

### Knowledge Gaps for Future Research

#### Patient Outcomes

Although patient outcomes were the most commonly reported outcome (Figure 3), none of the included studies directly measured the effect of CCDS systems on sepsis morbidity. Surviving sepsis is associated with cognitive impairment, higher mortality rates across the life span, physical disability, and mental health issues [3,4,6,7,9]. This not only substantially reduces the quality of life of survivors of sepsis but also presents an enormous financial burden on both patients and health care systems [5,8,171,172]. Reducing sepsis morbidity rates through CCDS use would be extremely valuable for patient health and quality of life and in mitigating personal and health care–related costs. Consequently, it is highlighted as a clear gap in the evidence base.

#### Usability and Cost Outcomes

We identified inadequate investigation of CCDS-related usability and cost outcomes, with most included studies focusing on clinical outcomes. The ability of a user to successfully operate a clinical information system is critical to the success of a system [25,173-175]. This is accentuated in the busy hospital environment, where medical providers are often time poor and carry enormous mental burdens [21,42]. Of particular concern in sepsis CCDS systems is the occurrence of alert fatigue [176,177]. Alert fatigue refers to when clinicians become desensitized to clinical alerts and consequently ignore or turn off alarm systems, potentially missing real sepsis cases [176,177]. This can have serious implications for patient outcomes. Strategies to ensure good CCDS system usability include incorporating human factor design elements, integrating CCDS system sepsis workflows into current medical emergency clinical pathways, and linking CCDS systems with existing clinical deterioration policies [42,178-180]. Only 11.3% (14/124) of the studies we investigated included usability outcomes, with only 14% (2/14) of these studies [70,93] evaluating alert fatigue. This represents a clear gap in the current literature for further research to support the successful implementation of appropriate, usable, and effective CCDS systems for early sepsis detection in hospitals.

In addition, very few studies investigated cost outcomes of CCDS system implementation. Sepsis is an extremely expensive condition to treat [171]. It has been reported to cost more than US $20 billion annually and is listed as the most financially costly condition in US hospitals [172]. Sepsis-related costs can range from extensive hospital costs during acute treatment to high long-term treatment and rehabilitation costs in survivors of sepsis [3,171,181]. Determining the cost-effectiveness of sepsis CCDS systems would assist in establishing the financial feasibility of implementation in hospitals and support widespread implementation.

#### Study Design and Generalizability

Few studies applied robust study designs such as randomized controlled trials, interrupted time series, stepped wedge clusters, and controlled trials. Future research in this area should attempt to use more rigorous methodology to present stronger evidence.

Approximately three-quarters of the included journal articles were conducted in the United States (Multimedia Appendix 6), limiting generalizability to other settings. A recent study demonstrated that the bulk of the sepsis burden is in countries with a low, low-middle, or middle sociodemographic index [2]. Future studies investigating the use of CCDS systems for adult sepsis inpatient identification should be encouraged to examine trends in countries outside the United States. In particular, CCDS systems should be evaluated in low- to middle-income countries when possible, given the limited availability of electronic health care technology in such regions.

### Reporting and Transparency

A large proportion of studies did not specify important study design, CCDS system, and main outcome details (Tables 1-3). An unexpectedly high number of journal articles did not report these details nor did most conference abstracts; however, this is more understandable given word limit constraints. Of particular concern is that almost two-thirds of the included journal articles were found to have an average or poor clarity of outcome reporting (Figure 4). None of the studies included in this review published the use of reporting guidelines, likely because of many journals not specifically requiring it. Overall, we found that the quality of reporting is low and identified a need for improved reporting and transparency throughout the literature.

### Strengths and Limitations

This scoping review comprehensively canvassed the literature investigating knowledge-based implemented CCDS systems for early sepsis detection in adult hospital patients. Its strength lies in this breadth of coverage and the wide range of study elements examined. The review followed the PRISMA-ScR expansion [28] guidelines, the Joanna Briggs Institute Reviewer’s Manual [27], and the framework presented by Arksey and O’Malley [29].

A limitation of this scoping review is that it only included studies written in English or had English translations readily available. Furthermore, only a sample of the charted data was double-checked by a second reviewer, potentially resulting in a higher error margin. However, the data charting forms were well structured, and any issues occurring during charting were fully discussed among the research team to reach consensus.

### Conclusions

This review highlights the extensive variability in the design, outcomes, and system characteristics in studies investigating the use of CCDS for the early detection of sepsis in adult inpatients. This heterogeneity can be largely attributed to the considerable complexity of sepsis, CCDS software, and the hospital environment. Our findings have identified clear gaps in the current literature, with few studies investigating CCDS system usability, cost, or the effects on patient morbidity. There are limited studies conducted outside the United States or with robust study designs. Our findings have illustrated frequent poor reporting of CCDS system information and study outcomes. It is critically important for future research to close these knowledge gaps, ensuring comprehensive evaluation of these rapidly emerging sepsis CCDS systems.

## Appendix

Multimedia Appendix 1 PRISMA-ScR (Preferred Reporting Items for Systematic Reviews and Meta-Analyses extension for Scoping Reviews) checklist.

Multimedia Appendix 2 Final MEDLINE search strategy.

Multimedia Appendix 3 Adjustments made to data charting form.

Multimedia Appendix 4 Definitions of groups combining multiple subgroups.

Multimedia Appendix 5 Main study characteristics.

Multimedia Appendix 6 Number of journal articles by country.

Multimedia Appendix 7 Main outcomes and outcome categories in gray literature.

Multimedia Appendix 8 Types of mortality reported in journal articles.

## Abbreviations

CCDS: computerized clinical decision support
ICU: intensive care unit
LILACS: Latin American and Caribbean Health Sciences Literature
PQDT: ProQuest Dissertations and Theses Global
PRISMA: Preferred Reporting Items for Systematic Reviews and Meta-Analyses
PRISMA-ScR: Preferred Reporting Items for Systematic Reviews and Meta-Analyses extension for Scoping Reviews
SIRS: systemic inflammatory response syndrome
