# Supplementary material for: Computerized Clinical Decision Support Systems for the Early Detection of Sepsis Among Adult Inpatients: Scoping Review
Source: J Med Internet Res. 2022 Feb 23;24(2):e31083. doi: 10.2196/31083 (PMC8908200; doi:10.2196/31083)
Supplement: Multimedia Appendix 2 [file jmir_v24i2e31083_app2.pdf]

Multimedia appendix 2: Final MEDLINE search strategy

1. sepsis/ or neonatal sepsis/ or shock, septic/ or systemic inflammatory response syndrome/

2. ("systemic inflammatory response syndrome" or sepsi* or SIRS or septi* or "septic shock").ti,ab.

3. 1 or 2

4. decision making, computer-assisted/ or diagnosis, computer-assisted/ or medical records systems, computerized/ or electronic health records/ or medical informatics/ or medical informatics applications/ or hospital information systems/ or Decision Support Systems, Management/ or Therapy, Computer-Assisted/ or telemedicine/ or telepathology/ or internet-based intervention/ or communications media/ or computers, handheld/ or smartphone/ or mobile applications/ or cell phone/ or text messaging/ or decision support systems, clinical/ or health information systems/ or Software/ or algorithms/ or electronics/ or electronics, medical/ or Automation/ or decision support techniques/ or clinical decision-making/ or clinical alarms/ or clinical decision rules/ or Point-of-Care Systems/ or biomedical technology/

5. ("health information systems" or CDS or CDSS or "knowledge-based" or "rule-based" or EHR or eHealth* or mHealth* or "mobile phone*" or "cell phone*" or "smart phone*" or software or algorithm or electronic or automat* or digital or computer* or computer-aided or computer-assisted or "clinic* decision support" or "best practice alert*" or "database query" or "clinical alert*" or "clinical alarm*" or "decision support").ti,ab.

6. exp artificial intelligence/ or exp machine learning/ or exp deep learning/ or exp support vector machine/ or exp neural networks, computer/

7. (artificial intelligence or deep learning or machine learning or support vector machine).ti,ab.

8. 6 or 7

9. 4 or 5

10. (screening or diagnos* or detect* or recog* or alert or alarm or triag* or identif* or surveillance or prognosis or "early warning system" or monitor* or warn* or tool).ti,ab.

11. diagnosis/ or early diagnosis/ or prognosis/ or Monitoring, Physiologic/

12. 10 or 11

13. 3 and 9 and 12

14. 13 not 8

15. exp animals/ not humans.sh.

16. 14 not 15

17. Limit 16 to english language
