# Supplementary material for: Computerized Clinical Decision Support Systems for the Early Detection of Sepsis Among Adult Inpatients: Scoping Review
Source: J Med Internet Res. 2022 Feb 23;24(2):e31083. doi: 10.2196/31083 (PMC8908200; doi:10.2196/31083)
Supplement: Multimedia Appendix 3 [file jmir_v24i2e31083_app3.pdf]

Multimedia appendix 3 – Adjustments made to data charting form

1. Fields added to the form:
    a. Whether the study was funded, including the funding source (i.e. commercial/non-commercial)
    b. What the study length in months and study start year were
    c. The number of sites, notes on the sites, and the number of participants
    d. What the publication type was (e.g. journal article vs. conference abstract)
    e. Whether the study followed any reporting guidelines. We also added 'clarity of outcomes reporting'. Studies were categorized as having 'good' clarity of outcome reporting if they specified the primary outcomes, the outcome analysis method, and the outcome measure definitions, and 'poor' clarity if the outcomes were not clearly described or there was a substantial reporting discrepancy between the methods and results. Studies were categorized as having 'average' clarity if they fulfilled some criteria of both good and poor.
    f. Whether the study reported the comorbidities of the included population
    g. What the EHR system is
    h. Whether the alert was run silently (i.e., the system was implemented but alerts were not delivered to clinicians) or was live (i.e., alerts are delivered).
    i. Whether an outcome was a primary outcome and what the analysis method was.
    j. We only charted data on the 'gold standard' sepsis definition used from studies looking at sepsis identification as an outcome.
2. Fields removed from form:
    a. We did not collect the study power
    b. Location of CCDS implementation within the hospital electronic infrastructure.
3. We used a flowchart to categorize the study design for each study, presented in figure 1 below. The flowchart was designed and piloted prior to data charting. We retrospectively removed a temporal element (prospective or retrospective) from the principal study type to simplify data charting. For example, 'prospective single cohort' simply became 'single cohort'.
4. We adjusted the definition of the usability outcome category to be the "extent to which a system, product or service can be used by specified users to achieve specified goals with effectiveness, efficiency, and satisfaction in a specific context of use" as defined by the International Organisation for Standardization (ISO) in ISO 9241-11:2018 section 3.1.1[1]. Accordingly, we reported usability outcomes into either CCDS system effectiveness, efficiency, or satisfaction with the CCDS system. We required usability outcomes to be investigated from the end-user perspective (e.g., clinicians).

Figure 1: Principal study categorization flow diagram

This flow chart was designed using reference material provided in appendix B of an Agency for Healthcare Research and Quality report[2] and articles by Ranganathan & Aggarwal[3-7].

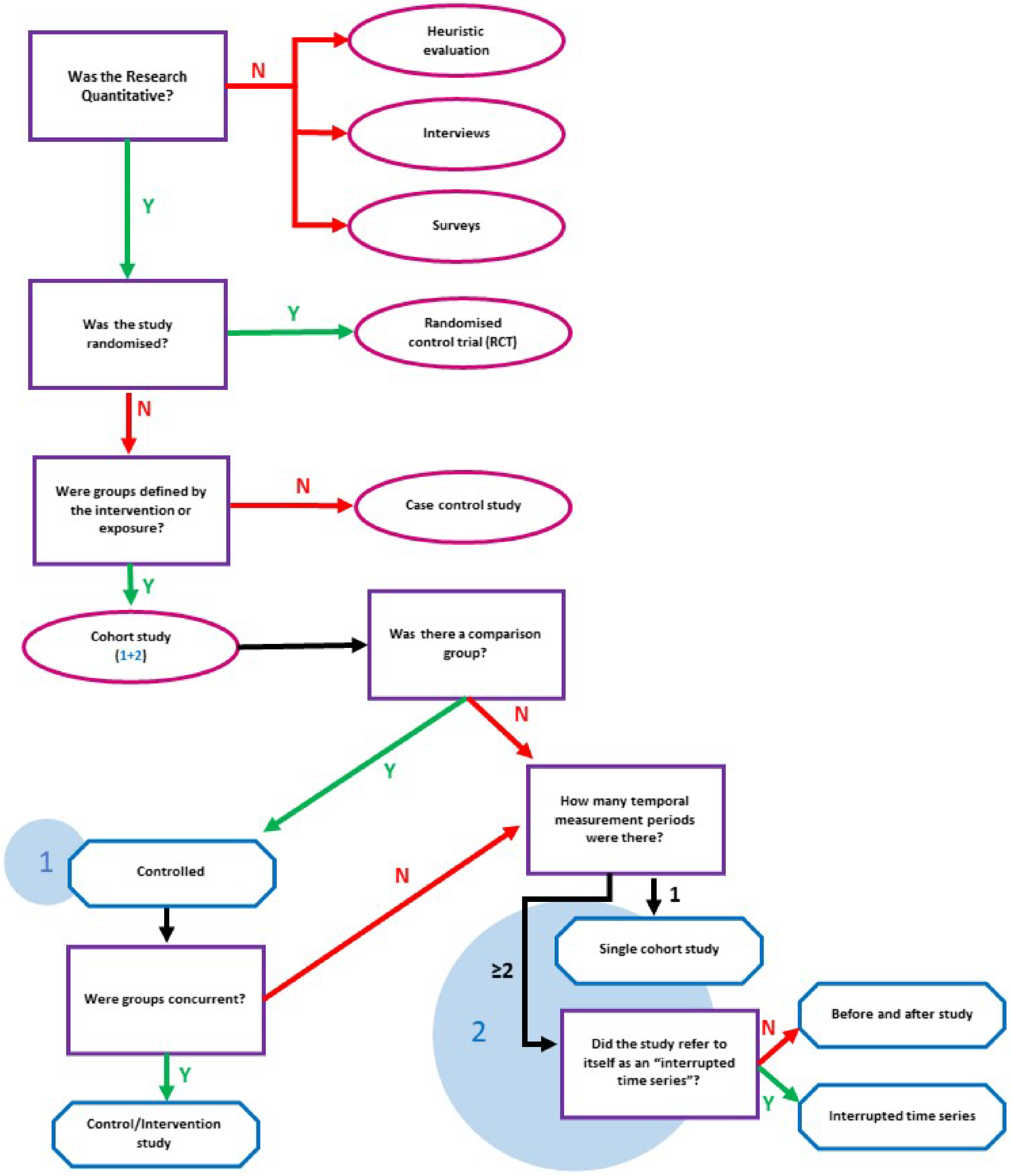

References

1. International Organization for Standardization (ISO). *Ergonomics of human-system interaction — Part 11: Usability: Definitions and concepts.* Standard No. 9241-11:2018(en). Available at: https://www.iso.org/obp/ui/#iso:std:iso:9241:-11:ed-2:v1:en [Accessed 26-02-2021]
2. Viswanathan M, Berkman N, Dryden D, Hartling L. *Assessing risk of bias and confounding in observational studies of interventions or exposures : further development of the RTI item bank.* In: AHRQ Methods for Effective Health Care. Rockville (MD): Agency for Healthcare Research and Quality (US); 2013.
3. Ranganathan P, Aggarwal R. Study designs: Part 1 – An overview and classification. *Perspectives in Clinical Research.* 2018;9(4):184-186. PMID:30319950
4. Aggarwal R, Ranganathan P. Study designs: Part 2 – Descriptive studies. *Perspectives in Clinical Research.* 2019;10(1):34-36. PMID:30834206
5. Ranganathan P, Aggarwal R. Study designs: Part 3 - Analytical observational studies. *Perspectives in Clinical Research.* 2019;10(2):91-94. PMID:31008076
6. Aggarwal R, Ranganathan P. Study designs: Part 4 – Interventional studies. *Perspectives in Clinical Research.* 2019;10(3):137-139. PMID:31404185
7. Aggarwal R, Ranganathan P. Study designs: Part 5 – interventional studies (II). *Perspectives in Clinical Research.* 2019;10(4):183-186. PMID:31649869
