# Supplementary material for: Computerized Clinical Decision Support Systems for the Early Detection of Sepsis Among Adult Inpatients: Scoping Review
Source: J Med Internet Res. 2022 Feb 23;24(2):e31083. doi: 10.2196/31083 (PMC8908200; doi:10.2196/31083)
Supplement: Multimedia Appendix 4 [file jmir_v24i2e31083_app4.pdf]

Multimedia appendix 4: Definitions of groups combining multiple subgroups

Table 1 – Study context and design

Conference abstract = combines the conference abstract, research showcase, conference abstract protocol, and clinical trial groups

Journal article = combines the journal article and plenary paper groups

Surveys/focus groups/heuristics = combines the questionnaires, interviews, focus groups and heuristic evaluation groups

Before-after = combines the controlled before-after and before-after (not controlled) groups

ICU = combines the burn ICU, ICU, medical ICU (MICU), surgical (SICU), surgical, trauma and burn ICU (STBICU), ED & ICU groups

Hospital-wide = combines the hospital-wide, and hospital-wide (not-ICU) groups. Note: if the study setting was not explicitly stated it was assumed that it was hospital-wide.

Inpatient wards = combines the acute care wards, inpatient wards, acute care wards (not ICU or ED), inpatient wards (not critical care), inpatient wards (not ED or ICU), inpatient wards (not ED), inpatient wards (not ICU), internal medicine department, medical-surgical units, non-critical care wards, ICU & inpatient wards, ICU & inpatient wards (not ED), ED & inpatient wards (not ICU), and ED & inpatient wards groups

Specific ward = combines the specific ward and cancer center groups

Figure 2 - Number of references by publication type and year published

Conference abstract = combines the conference abstract, research showcase, conference abstract protocol, and clinical trial groups

Journal article = combines the journal article and plenary paper groups

Figure 3 – Proportion of studies reporting each outcome category

Conference abstract = combines the conference abstract, research showcase, conference abstract protocol, and clinical trial groups

Journal article = combines the journal article and plenary paper groups

Figure 4 – Clarity of outcome reporting in the studies

Conference abstract = combines the conference abstract, research showcase, conference abstract protocol, and clinical trial groups

Journal article = combines the journal article and plenary paper groups

Table 2 - Main outcomes and outcome categories in journal articles AND Multimedia appendix 8 - Main outcomes and outcome categories in grey literature

Cost

Cost = combines the hospital cost, hospital charges for sepsis patients, total direct cost, facility utilization costs, pharmacy costs, laboratory tests costs, supplies costs, imaging costs, laboratory management costs, other costs, costs per stay, variable indirect costs, total cost of care, fixed direct costs, fixed indirect costs, variable direct cost, and total cost outcomes

Cost effectiveness/savings = combines the cost-effectiveness, cost savings, and cost & utilization outcomes

Patient outcomes

ICU admission = combines the ICU admission, ICU admission <48h after alert, unplanned ICU admission within 24 hours of admission to hospital, ICU admission within 12hrs of alert, ICU admission < 6h after alert, ICU admission <24h after alert, and ICU admission within 48hr of alert outcomes

LOS = combines the LOS (ICU), LOS (hospital), LOS (ICU) among survivors, LOS (hospital) among survivors, LOS (>72 hours), LOS (non-ICU), LOS (ED), LOS (prolonged >=7 days), LOS, LOS (hospital) after alert, and LOS (above predicted) outcomes

Mortality = combines the mortality (in-hospital), mortality (in-hospital post NP-RRT interaction), mortality (admitted inpatients with sepsis), mortality, mortality (sepsis-related), hospital mortality rate, mortality (in-hospital 30-day), mortality index (sepsis-related), unadjusted mortality, adjusted mortality, mortality (ED admitted patients with sepsis), predictors of mortality (in-hospital), mortality (30-day) prediction, mortality (in-hospital) prediction, mortality (28-day in-hospital) prediction, mortality (1-day) prediction, mortality (5-day) prediction, mortality (10-day) prediction, mortality (60-day) prediction, mortality (hospital), mortality (inpatient), mortality (ICU), mortality (Health care facility-onset Clostridium difficile infections), mortality (30-day in-hospital all-cause), mortality (14 day), mortality (respiratory failure related), mortality (sepsis-related inpatient), mortality (sepsis), mortality (90-day), mortality (28-day), mortality index, mortality (in-hospital all-cause), mortality rate, risk-adjusted mortality index, mortality (30-day all-cause), mortality (all-cause 7 day), mortality (all-cause 14 day), mortality (30-day), mortality (within 30 days of alert), mortality (ICU admitted), mortality (sepsis-related in-hospital), mortality (severe sepsis-related), mortality (septic shock-related), and mortality within 48hr of alert outcomes

Other (patient outcomes) = combines the 28-day hospital-free days, outcome (either adverse or positive), composite of death/ICU admission, composite of in-hospital death/hospice referral, serum lactate >= 4mmol/L (admitted patients), transfer to higher care level, sepsis vs. severe sepsis/septic shock encounters, sepsis screening vital sign abnormality characteristics, septic patients outcome, discharged to hospice, enrolment into study, health care facility-onset *clostridium difficile* infection cases, hypotension-free days, ventilator-free days, hospital discharge routes, 30-day hospital readmission, proportion of patients with positive screens, mean sepsis score, maximum sepsis score, number of alerts, transfers to higher levels of care, hospital disposition (includes discharge characteristics & in-hospital mortality), APACHE II score, discharge outcome, lactate concentration =< 2mmol/L, lactate concentration >= 4mmol/L, positive alert (both patient & procedure characteristics), *Clostridioides difficile* cases, identification of eligible patients for study, rapid response team < 6h after alert, mortality or inpatient hospice transfer, discharge to home, composite of ICU admission rapid response team (RRT) activation & mortality, sepsis incidence, severe sepsis incidence, septic shock incidence, readmission rate, serious adverse event within 48hr of alert, rapid response within 48hr of alert, code call within 48hr of alert, composite outcome: all-cause in-hospital death and/or ICU

admission, rapid response for septic shock, number of rapid response team (RRT) activations, number of code blues, rapid response team (RRT) escalation, critical care medicine consult escalation, and rapid response team activation outcomes

Sepsis identification = combines the sepsis prediction, early recognition of sepsis, sepsis identification, confirmed sepsis cases, severe sepsis identification, confirmed diagnosis of severe sepsis or septic shock, sepsis diagnosis, sepsis recognition, severe sepsis diagnosis, sepsis prediction accuracy, sepsis diagnosis at discharge, CLABSI rates, severe sepsis/septic shock identification, shock development, discharges with a sepsis septic shock or severe sepsis code, discharges with a sepsis code, discharges with a septic shock code, discharges with a severe sepsis code, sepsis diagnosis (number), sepsis recognition by house staff following alert, final hospital infection diagnosis, ED infection diagnosis, sepsis category diagnosis (ICD 9/10 category), severe sepsis detection, severe sepsis & septic shock identification, severe sepsis & septic shock recognition, any sepsis-related diagnosis, severe sepsis/septic shock, sepsis discharge diagnosis, sepsis ratio of observed to expected, PPV for sepsis identification, severe sepsis & septic shock detection, and sepsis identification accuracy outcomes

<u>Sepsis treatment/management</u>

Antibiotics = combines the time to first antibiotic, time to antibiotics order from ED arrival, time to antibiotics administration from ED arrival, antibiotics administered within 3hrs, time to antibiotic administration, antibiotic administration, antibiotics within 1hr of alert, time to antibiotics from alert, antibiotics administered within 3hrs of alert, antibiotics within 1hr of alert, new antibiotics within 24hr following alert, time of new antibiotics, time from protocol order to new antibiotic administration, new antibiotics ordered within 3 hours, time of antibiotic administration, time to antibiotics, antibiotics administered within 3hr of triage, appropriate antibiotics for source, antibiotics administered within 3hrs of admission, antibiotics administered, time to first antibiotics from arrival, antibiotics within 1hr of ED arrival, administration of broad-spectrum antibiotics, cefepime administration, levofloxacin administration, ceftriaxone administration, ciprofloxacin administration, piperacillin-tazobactam administration, imipenem-cilastatin administration, ertapenem administration, aztreonam administration, clindamycin administration, cefepime + imipenem or cilastatin + aztreonam, time to new antibiotics from alert, time to new/changed antibiotic administration, antibiotic administration within 24hr of SIRS onset, broad-spectrum antibiotic administration with 24hours of SIRS onset, community antibiotic administration with 24hours of SIRS onset, broad-spectrum antibiotics ordered, time to antibiotic administration following alert, time to antibiotic administration from alert, time to first antimicrobials, time to antibiotics from admission, adequate antibiotics, early antibiotic administration, inappropriate antibiotic administration, antibiotic escalation within 12hrs, time to first antibiotics upon ED arrival, antibiotics administered within 3hrs of ED presentation, daily antibiotic use, antibiotic therapy length, time from severe sepsis/septic shock onset to first antibiotics, sepsis antibiotic order <3 h after alert, antibiotic initiation/change in current antibiotics, appropriate antibiotic administration, time to antibiotics from diagnosis, and new broad-spectrum antibiotics ordered up to 48hr before or 6hr after alert outcomes

Blood culture = combines the time to culture from ED arrival, blood culture ordered, blood culture pre-antibiotics, time to blood culture, blood culture <3 h after alert, time of blood culture, blood culture obtained, time to blood culture from alert, and time to blood culture collection outcomes

Fluids = combines the time to resuscitation, fluids delivered, fluids (≥ 20mL/kg) and vasopressor use in first 6hrs, 20mL/kg of fluids ordered, cumulative volumes of fluid administered, IV fluids within 3 hours of alert, at least 30mL/kg IV fluids within 3hrs, at least

2L of fluids within 3hrs, amount & time of crystalloid administration, 2L of fluids administered within 2hr of triage, ≥30mL/kg IV fluids, time to 30mL/kg fluid administration, time to fluid bolus from arrival, 6-hour fluid administration, daily fluid administration, time to IV fluids from alert, appropriate fluid resuscitation, fluid administration within 12hrs, ≥500 mL IV bolus order <3 h after alert, and ≥500mL fluid within 30mins outcomes

Lactate = combines the time to first lactate from ED arrival, time to first lactate, serum lactate ordered, lactate measured within 6hrs, lactate ordered, serum lactate ordered in ED, serum lactate ordered after admission, lactic acid order <3 h after alert, time of lactate, serum lactate reordered if >4mmol/L, lactate measured, time to lactate, lactate measurement, time to lactate collection from alert, time to lactate collection, lactate obtained within 3hrs of alert, and at least 2 lactate tests within 6hrs of ED presentations outcomes

Other (sepsis treatment/management) = combines the physician ordering changes, IVC ultrasound use, time to ICU admission from alert, timeliness of sepsis alert, time between alert and onset of suspected infection, mechanical ventilation, vasopressor use, MAP raised and remained > 65 mmHg, CVP≥ 8mmHg achieved <6hr, CVP≥ 8mmHg achieved <24hr, CVP≥8mmHg achieved >24hr, time to CVP, ScvO2 ≥ 70% achieved <6hr, ScvO2 ≥ 70% achieved <24hr, ScvO2 ≥ 70% achieved >24hr, time to ScvO2, appropriate use of SSRT, appropriate use of ICU consult, time to onset of severe sepsis, team notification following alert, time of BPA, response to BPA, alert timing, timeliness of alert, number of sepsis cases managed, improvement of sepsis alert criteria, appropriateness of care level for patients, sepsis screening time, non-invasive cardiac output monitoring, inpatient sepsis screening, time to alert acknowledgement from alert, time to sepsis identification, meets resuscitation end points, ventilator duration, time between alarm and septic event, days on inotropic support, days on ventilator, CVC placed if lactate >4, CVP measurement, ScvO2 measurement, time to sepsis resuscitation, time to alert acknowledgement, alert response rate, time to hospital admission from arrival, time to pressor use, testing for *clostridium difficile* infection, rate of intubation, time to chart open after MEWS ≥ 5 (alert 1), education pre-test & post-test scores, VSA compliance, number of patients screened, number of screens per unique patient, time from alert to any sepsis-related intervention, number of alerts, time alert from admission, time from ED arrival to alert, time from triage to alert, time from room placement to alert, chest radiograph performed pre-admission, time to pre-admission chest radiograph, sepsis identification timeliness, time to ICU referral from alert, administration of interventions within 12hrs of alert, oxygen therapy within 12hrs, microbiological tests within 12hrs, radiographic imaging within 12hrs, intubation, time to alert from admission, microbiological diagnosis rates, radiological diagnosis rates, average time per patient to complete study enrolment, average time per eligible patient to complete study enrolment, blood gas order <6 h after alert, BMP or CBC order <6 h after alert, vasopressor order <6 h after alert, bronchodilator administration <6 h after alert, transfusion order <6 h after alert, AV node blocker order <6 h after alert, loop diuretic order <6 h after alert, CXR <6 h after alert, CT head/chest/abdomen <6 h after alert, cardiac monitoring <6 h after alert, time to first ICU admission after alert, naloxone order <6h after alert, time to diagnosis from alert, nurse alert response time, and physician alert response time outcomes

Sepsis bundle/protocol compliance = combines the sepsis bundle compliance, number of sepsis protocols initiated, time to sepsis bundle completion from ED arrival, 3-hour bundle compliance, order set usage, sepsis bundle compliance (SEP-1), order set compliance, time to bundle completion from alert, time to bundle completion from alert acknowledgement, timeliness of alert acknowledgement if time to bundle completion was achieved, provider documentation meeting CMS criteria for SEP-1, time to bundle compliance, adherence to SOP,

adherence to anti-infective SOP, vital sign documentation compliance, and time from first concern for sepsis to bundle compliance outcomes

Usability

Effectiveness = combines the effectiveness of system (provider), effectiveness of system (other clinicians), effectiveness of system (nurses), effectiveness of system, effectiveness of system (heuristics evaluation), and effectiveness of system (medical trainees) outcomes

Efficiency = combines the efficiency of system (provider), efficiency of system (other clinicians), efficiency of system (nurses), efficiency of system (heuristics evaluation), and efficiency of system (medical trainees) outcomes

Satisfaction = combines the satisfaction of system (provider), satisfaction of system (other clinicians), satisfaction of system (nurses), satisfaction of system (medical trainees), and satisfaction of system (nurses & physicians) outcomes

Table 3 - Computerized clinical decision support (CCDS) characteristics

Conference abstract = combines the conference abstract, research showcase, conference abstract protocol, and clinical trial groups

Journal article = combines the journal article and plenary paper groups

CCDS type

Commercial = References who implemented only a commercial CCDS, as well as references that implemented both a homegrown and commercial CCDS simultaneously.

Silent or Live?

Both = Both silent and live at different stages of the study, either concurrently, before/after, or for separate outcomes

CCDS criteria

SIRS = combines the SIRS and modified SIRS groups

SIRS + Organ dysfunction = combines the SIRS + organ dysfunction and modified SIRS + organ dysfunction groups

SIRS + Other = combines the SIRS + Other, SIRS + MEWS + NEWS + shock, SIRS + MAP, SIRS + (Organ dysfunction OR fluid nonresponsive hypotension), SIRS + Organ dysfunction + shock, SIRS + sBP, SIRS + sBP + serum creatinine, SIRS + shock, SIRS + sign of infection + sBP, SIRS + sign of infection + shock, SIRS + Organ dysfunction + Sign of infection, and SIRS + sign of infection groups

Other = combines the other, abnormal vital signs, organ dysfunction, abnormal vital signs & laboratory results, blood cultures & vasopressor ordered/administered within 24 hours of each other, nursing assessment + abnormal vital signs, sepsis-2, sepsis-3, qSOFA + sign of suspected infection + SOFA, adjusted MEWS-SRS, Burn6, MEWS, M2SEWS, MEDS, MEWS-SRS, NEWS, REMS, SCS, and SOFA groups

Related interventions

Clinical protocol = combines the clinical pathway, care plan/bundle, clinical pathway/sepsis protocol, emphasis on vital sign documentation, improved triage system, increased vital sign

screening, power plans, prompted documentation, screening protocol, standing orders, and treatment policy groups

Education/staff resources = combines the education (alert), education (sepsis & response), education (sepsis documentation & coding), help line, intranet resources, and reminders groups

Electronic/infrastructure changes = combines the automated time zero algorithm, data collection system/warehouse, database to manage compliance & timely treatment, patient dashboard, sepsis care patient record, sepsis dashboard, and stocked medication dispensers' groups

Response/Leadership team = combines the response team, sepsis leadership committee, sepsis program leader/leadership committee/team, and surveillance team groups

Order sets = combines the order set (antibiotics), order set (fluids), order set (pathology), and order set (general) groups

Feedback = combines the audit & feedback, progress updates, and updates on bundle compliance groups

Responding personnel

Nurse = combines the nurse, ED charge nurse, critical care nurse, intensive care nurse, nurse practitioner, triage nurse, and tele-ICU nurse groups

Other clinicians = combines the clinician, provider, ED clinician, physician assistant, rapid response team physician, ED provider, physician, doctor, ED physician, and intensivist groups

Response team = combines the rapid response team, nurse-practitioner rapid response team, and medical team groups

Study coordinator = combines the quality improvement specialist, rapid response coordinator, sepsis coordinator, study coordinator, and study coordinator/investigator groups

Other = combines the sepsis surveillance personnel, licensed independent practitioner (LIP), ICU staff, pharmacist, house staff, technician, ED floor staff, telemetry personnel, and hospital telephone service groups

Alert delivery

Electronic patient record = combines the EHR, electronic ED patient chart, patient record, pop-up notification, medical record flow sheet, BPA, digital order communications system, and electronic prompt groups

Patient dashboard/working list = combines the Patient dashboard/viewer, nurse work list, ED dashboard, nurse tracking board, ICU display, and tele-ICU census screen groups

Other = combines the database query, email, mobile-phone, and printer groups
