# Supplementary material for: Computerized Clinical Decision Support Systems for the Early Detection of Sepsis Among Adult Inpatients: Scoping Review
Source: J Med Internet Res. 2022 Feb 23;24(2):e31083. doi: 10.2196/31083 (PMC8908200; doi:10.2196/31083)
Supplement: Multimedia Appendix 5 [file jmir_v24i2e31083_app5.pdf]

Multimedia Appendix 5: Main study characteristics

Author (Year)	Type of publication	Country	Principal study type	Setting	Number of sites	Number of participants	Population	CCDS type	CCDS sepsis criteria	Outcome category
Aakre (2016)[40]	Clinical Trial	Unspecified	single cohort	ICU	NR	215	Adult	Unspecified	SOFA	Sepsis Treatment/Management, Usability
Aakre (2017)_1[41]	Journal Article	United States	single cohort	ICU	2	215	Age not specified	Homegrown	SOFA	Patient Outcomes
Aakre (2017)_2[42]	Journal Article	United States	Interview	ICU	NR	12	Adult	Homegrown	SOFA	Usability
Acquah (2014)[43]	Conference Abstract	United States	Not enough information to determine	ED	1	NR	Age not specified	Homegrown	Unspecified	Sepsis Treatment/Management
Afshar (2019)[44]	Journal Article	United States	before/after (not controlled)	Hospital-wide	1	13877	Adult	Homegrown	MEWS	Patient Outcomes, Sepsis Treatment/Management, Cost
Alsolamy (2014)[45]	Journal Article	Saudi Arabia	single cohort	ED	1	49838	Adult	Homegrown	Organ dysfunction, SIRS + Organ dysfunction	Patient Outcomes, Sepsis Treatment/Management
Amland (2016)_1[46]	Journal article	United States	single cohort	Hospital-wide	5	6200	Adult	Commercial	SIRS, SIRS + Organ dysfunction	Patient Outcomes, Sepsis Treatment/Management
Amland (2016)_2[47]	Journal Article	United States	single cohort	Hospital-wide (not-ICU)	1	16527	Adult	Commercial	SIRS, SIRS + Organ dysfunction	Patient Outcomes
Amland (2018)_1[48]	Journal Article	United States	single cohort	Hospital-wide	8	17044	Adult	Commercial	SIRS, SIRS + Organ dysfunction	Patient Outcomes, Sepsis Treatment/Management
Amland (2018)_2[49]	Journal Article	United States	single cohort	Hospital-wide	8	75958	Adult	Commercial	SIRS, SIRS + Organ dysfunction	Patient Outcomes, Sepsis Treatment/Management
Arabi (2017)[50]	Journal Article	Saudi Arabia	before/after (not controlled)	ED	1	1330	Adult	Homegrown	Organ dysfunction, SIRS + Organ dysfunction	Patient Outcomes, Sepsis Treatment/Management
Austrian (2018)[51]	Journal article	United States	interrupted time series (not controlled)	ED	1	2144	Adult	Homegrown	Other, SIRS	Patient Outcomes, Sepsis Treatment/Management
Bansal (2018)[52]	Journal Article	United States	single cohort	ED	1	27106	Age not specified	Unspecified	SIRS, Unspecified	Patient Outcomes, Sepsis Treatment/Management
Becker (2018)[53]	Conference Abstract	United States	before/after (not controlled)	ED	1	903	Age not specified	Unspecified	Other	Patient Outcomes, Sepsis Treatment/Management
Benson (2014)[54]	Journal Article	United States	before/after (not controlled)	medical-surgical units	1	239	Age not specified	Homegrown	Other + SIRS	Patient Outcomes, Sepsis Treatment/Management, Cost
Berger (2010)[55]	Journal Article	United States	before/after (not controlled)	ED	1	5796	Adult	Homegrown	SIRS	Patient Outcomes, Sepsis Treatment/Management
Biltoft (2012)[56]	Conference Abstract	United States	before/after (not controlled)	Hospital-wide (not-ICU)	1	NR	Age not specified	Unspecified	SIRS	Patient Outcomes, Sepsis Treatment/Management, Cost
Bradley (2019)[57]	Conference Abstract	United States	before/after (not controlled)	Specific ward	3	NR	Age not specified	Homegrown	MEWS	Patient Outcomes
Brandt (2015)[58]	Journal Article	United States	single cohort	Hospital-wide	1	NR	Adult	Homegrown	SIRS + Organ dysfunction, SIRS + sign of suspected infection	Patient Outcomes, Sepsis Treatment/Management

Author (Year)	Type of publication	Country	Principal study type	Setting	Number of sites	Number of participants	Population	CCDS type	CCDS sepsis criteria	Outcome category
Brown (2016)[59]	Journal Article	United States	single cohort	ED	1	58603	Adult	Homegrown	SIRS + MAP	Patient Outcomes
Buck (2014)[60]	Journal Article	United States	single cohort	Specific ward	3	617	Age not specified	Homegrown	SIRS + Organ dysfunction	Patient Outcomes
Carlbom (2014)[61]	Conference Abstract	Unspecified	single cohort	Acute care wards (not ICU or ED)	NR	NR	Age not specified	Homegrown	Other, SIRS	Patient Outcomes
Chanas (2019)[62]	Journal Article	United States	single cohort	Surgical, trauma, and burn ICU	1	97	Age not specified	Unspecified	Blood cultures & vasopressor ordered/administrated within 24 hrs of each other	Patient Outcomes, Sepsis Treatment/Management
Chang (2015)[63]	Conference Abstract	United States	single cohort	ICU	1	91	Adult	Homegrown	SIRS + Organ dysfunction	Patient Outcomes
Colorafi (2019)[64]	Journal Article	United States	single cohort	Hospital-wide	26		Adult	Commercial	SIRS, SIRS + Organ dysfunction	Patient Outcomes, Sepsis Treatment/Management
Comlekoglu (2020)[65]	Conference Abstract	Unspecified	controlled study	ED	1	16234	Age not specified	Unspecified	SIRS	Patient Outcomes, Sepsis Treatment/Management
Croft (2014)[66]	Plenary paper	United States	before/after (not controlled)	Surgical ICU	NR	184	Age not specified	Homegrown	MEWS-SRS	Patient Outcomes, Sepsis Treatment/Management
Danak (2014)[67]	Thesis	United States	single cohort	ED	1	20789	Adult	Unspecified	SIRS	Patient Outcomes, Sepsis Treatment/Management
Downing (2019)[68]	Journal article	Unspecified	RCT	Inpatient wards (not ICU)	1	1123	Adult	Homegrown	SIRS + Organ dysfunction + sign of suspected infection	Patient Outcomes, Sepsis Treatment/Management, Usability
Dumont (2013)[69]	Journal Article	United States	before/after (not controlled)	Hospital-wide	NR	NR	Age not specified	Homegrown	SIRS	Patient Outcomes, Sepsis Treatment/Management
Dziadzko (2016)[70]	Journal Article	United States	RCT	ICU	1	23	Adult	Unspecified	Unspecified	Sepsis Treatment/Management, Usability
Eck (2009)[71]	Conference Abstract	Unspecified	single cohort	ICU	1	176	Age not specified	Homegrown	SIRS + Organ dysfunction + shock	Patient Outcomes, Sepsis Treatment/Management
Ehrlichman (2014)[72]	Conference Abstract	Unspecified	single cohort	Specific ward	1	301	Age not specified	Commercial	SIRS, SIRS + Organ dysfunction	Patient Outcomes
Engineer (2016)[73]	Conference abstract protocol	United States	single cohort	ED	1	NR	Age not specified	Homegrown	Abnormal vital signs & laboratory results, SIRS	Sepsis Treatment/Management
Eren (2016)[74]	Conference Abstract	Turkey	single cohort	ICU	1	1384	Age not specified	Homegrown	SIRS + SBP	Patient Outcomes
Faisal (2019)[75]	Journal Article	United Kingdom	single cohort	ED	3	73851	Adult	Unspecified	NEWS	Patient Outcomes
Falimirski (2016)[76]	Conference Abstract	United States	single cohort	Non-critical care wards	2	313	Age not specified	Homegrown	modified SIRS	Patient Outcomes

Author (Year)	Type of publication	Country	Principal study type	Setting	Number of sites	Number of participants	Population	CCDS type	CCDS sepsis criteria	Outcome category
Fee (2014)[77]	Conference Abstract	United States	before/after (not controlled)	ED	1	NR	Adult	Unspecified	SIRS, SIRS + Organ dysfunction	Patient Outcomes, Sepsis Treatment/Management
Fogerty (2016)[78]	Conference Abstract	Unspecified	single cohort	Inpatient wards	NR	11894	Adult	Unspecified	modified SIRS	Patient Outcomes, Sepsis Treatment/Management
Fogerty (2019)[79]	Journal Article	United States	before/after (not controlled)	Internal Medicine Department	1	15739	Age not specified	Homegrown	SIRS + SBP + serum creatinine	Patient Outcomes
Gatewood (2015)[80]	Journal Article	United States	before/after (not controlled)	ED	1	NR	Age not specified	Homegrown	SIRS	Patient Outcomes, Sepsis Treatment/Management
Gerald (2011)_1[81]	Conference Abstract	Unspecified	single cohort	Hospital-wide (not-ICU)	NR	7247	Adult	Commercial	SIRS + Organ dysfunction	Patient Outcomes
Gerald (2011)_2[82]	Conference Abstract	United States	single cohort	Hospital-wide (not-ICU)	1	1560	Adult	Unspecified	SIRS + Organ dysfunction	Patient Outcomes
Gerald (2011)_3[83]	Conference Abstract	United States	single cohort	Hospital-wide (not-ICU)	1	625	Adult	Unspecified	SIRS + Organ dysfunction	Patient Outcomes
Ghanem-Zoubi (2011)[84]	Journal Article	Israel	single cohort	Internal Medicine Department	1	1072	Adult	Homegrown	MEDS, MEWS, REMS, SCS, SIRS	Patient Outcomes
Giesler (2014)[85]	Conference Abstract	United States	single cohort	ED	2	166	Adult	Unspecified	SIRS	Patient Outcomes, Sepsis Treatment/Management
Gille (2017)[86]	Journal Article	Germany	single cohort	Burn ICU	1	41	Adult	Commercial	SIRS	Patient Outcomes, Sepsis Treatment/Management
Gowda (2011)_1[87]	Conference Abstract	Unspecified	single cohort	ICU	NR	611	Age not specified	Unspecified	modified SIRS	Patient Outcomes
Gowda (2011)_2[88]	Conference Abstract	Unspecified	single cohort	ICU	NR	439	Age not specified	Unspecified	modified SIRS	Patient Outcomes, Sepsis Treatment/Management
Grek (2017)[89]	Journal Article	United States	before/after (not controlled)	ED	1	NR	Age not specified	Homegrown	SIRS, Unspecified	Patient Outcomes, Sepsis Treatment/Management
Guidi (2015)[90]	Journal Article	United States	Survey	Inpatient wards (not critical care)	1	232	Adult	Homegrown	SIRS + Organ dysfunction	Usability
Guirgis (2017)[91]	Journal Article	United States	before/after (not controlled)	Hospital-wide	1	3205	Adult	Homegrown	Adjusted MEWS-SRS	Patient Outcomes, Sepsis Treatment/Management, Cost
Harrison (2015)[92]	Thesis	United States	before/after (not controlled)	ED, ICU	3	158	Adult	Homegrown	SIRS + Organ dysfunction + sign of suspected infection	Patient Outcomes, Sepsis Treatment/Management
Harrison (2017)[93]	Journal Article	United States	controlled study	ICU	2	12	Adult	Homegrown	Unspecified	Sepsis Treatment/Management, Usability
Hayden (2016)[94]	Journal Article	Unspecified	before/after (not controlled)	ED	1	238	Adult	Homegrown	SIRS + sign of infection + SBP	Patient Outcomes, Sepsis Treatment/Management
Herasevich (2011)[95]	Journal Article	United States	before/after (not controlled)	ICU	3	8609	Age not specified	Homegrown	SIRS + sign of infection + shock	Patient Outcomes

Author (Year)	Type of publication	Country	Principal study type	Setting	Number of sites	Number of participants	Population	CCDS type	CCDS sepsis criteria	Outcome category
Hiensch (2017)[96]	Journal Article	United States	interrupted time series (not controlled)	Specific ward	4	NR	Adult	Homegrown	Other	Patient Outcomes, Sepsis Treatment/Management
Honeyford (2020)[97]	Journal Article	United Kingdom	before/after (not controlled)	Hospital-wide	3	21732	Adult	Commercial	SIRS, SIRS + Organ dysfunction	Patient Outcomes, Sepsis Treatment/Management
Hooper (2010)[98]	Conference Abstract	Unspecified	RCT	ICU	NR	77	Age not specified	Unspecified	modified SIRS	Patient Outcomes, Sepsis Treatment/Management
Hooper (2011)[99]	Conference Abstract	Unspecified	RCT	ICU	NR	10727	Age not specified	Unspecified	modified SIRS	Sepsis Treatment/Management
Hooper (2012)[100]	Journal Article	United States	RCT	Medical ICU	1	442	Age not specified	Unspecified	modified SIRS	Patient Outcomes, Sepsis Treatment/Management
Horton (2020)[101]	Journal Article	United States	interrupted time series (not controlled)	Acute care wards (not ICU or ED)	8	NR	Adult	Homegrown	MEWS	Patient Outcomes, Sepsis Treatment/Management, Cost
Huff (2019)[102]	Journal Article	Unspecified	Not enough information to determine	Specific ward	3	NR	Age not specified	Homegrown	NEWS, SIRS	Patient Outcomes, Sepsis Treatment/Management, Usability
Hughes (2019)[103]	Journal Article	United States	controlled before/after	Hospital-wide	3	4475	Adult	Unspecified	SIRS + Organ dysfunction	Patient Outcomes, Sepsis Treatment/Management, Cost
Jones (2015)[104]	Journal Article	United States	before/after (not controlled)	ICU, Inpatient wards (not ICU)	1	NR	Adult	Homegrown	Other	Patient Outcomes, Sepsis Treatment/Management, Cost
Judd (2014)[105]	Journal Article	United States	before/after (not controlled)	Hospital-wide	1	397	Adult	Unspecified	SIRS + Organ dysfunction	Patient Outcomes, Cost
Jukes (2013)[106]	Thesis	United States	interrupted time series (not controlled)	Hospital-wide	2	51315	Adult	Homegrown	SIRS + Organ dysfunction + sign of suspected infection	Patient Outcomes, Sepsis Treatment/Management
Jung (2018)[107]	Journal Article	United States	before/after (not controlled)	Surgical ICU	1	30	Age not specified	Homegrown	Other	Patient Outcomes, Sepsis Treatment/Management
Kangas (2019)[108]	Journal Article	United States	single cohort	Inpatient wards (not ICU)	1	2039	Adult	Homegrown	MEWS + NEWS + SIRS + shock combination, SIRS + shock	Patient Outcomes
Krieger (2013)[109]	Conference Abstract	United States	single cohort	ED	1	65043	Adult	Homegrown	Other	Patient Outcomes
Kurczewski (2015)[110]	Journal Article	United States	before/after (not controlled)	Hospital-wide	NR	60	Adult	Unspecified	modified SIRS	Patient Outcomes, Sepsis Treatment/Management
Lawson (2017)[111]	Thesis	United States	before/after (not controlled)	Inpatient wards (not ICU)	4	NR	Adult	Unspecified	Unspecified	Patient Outcomes, Sepsis Treatment/Management
Liang (2018)[112]	Conference Abstract	United States	controlled study	ED, Inpatient wards (not ICU)	NR	NR	Age not specified	Unspecified	MEWS, SIRS	Sepsis Treatment/Management
MacMillan (2019)[113]	Journal Article	Unspecified	before/after (not controlled)	ICU	1	160	Adult	Homegrown	SIRS	Patient Outcomes, Sepsis Treatment/Management, Cost

Author (Year)	Type of publication	Country	Principal study type	Setting	Number of sites	Number of participants	Population	CCDS type	CCDS sepsis criteria	Outcome category
Mann-Salinas (2014)[114]	Conference Abstract	Unspecified	single cohort	ICU	NR	47	Age not specified	Unspecified	Burn6	Patient Outcomes
Manteuffel (2013)[115]	Conference Abstract	United States	before/after (not controlled)	ED	1	94556	Adult	Unspecified	SIRS + sBP	Patient Outcomes
Martin-Rico (2014)[116]	Conference Abstract	Unspecified	single cohort	ED	1	37323	Adult	Homegrown	SIRS + Organ dysfunction	Patient Outcomes, Sepsis Treatment/Management
McCabe (2015)[117]	Conference Abstract	Unspecified	Not enough information to determine	Hospital-wide	NR	NR	Age not specified	Homegrown	M2SEWS	Patient Outcomes
McGrane (2010)[118]	Conference Abstract	Unspecified	single cohort	ICU	NR	327	Age not specified	Unspecified	modified SIRS	Patient Outcomes
McRee (2014)[119]	Journal Article	Unspecified	before/after (not controlled)	Specific ward	1	171	Adult	Unspecified	SIRS	Patient Outcomes
Meurer (2009)[120]	Journal Article	Unspecified	single cohort	ED	1	583	Adult	Homegrown	SIRS	Patient Outcomes, Sepsis Treatment/Management
Miller (2017)[121]	Journal Article	United States	Survey	Specific ward	5	151	Adult	Commercial	Abnormal vital signs & laboratory results	Usability
Muratore (2019)[122]	Journal Article	United States	controlled study	Hospital-wide (not-ICU)	1	10335	Adult	Homegrown	modified SIRS	Patient Outcomes, Sepsis Treatment/Management
Narayanan (2016)[123]	Journal Article	Unspecified	before/after (not controlled)	ED	1	214	Adult	Homegrown	SIRS, SIRS + (Organ dysfunction OR fluid nonresponsive hypotension)	Patient Outcomes, Sepsis Treatment/Management
Nelson (2011)[124]	Journal Article	Unspecified	before/after (not controlled)	ED	1	33460	Adult	Unspecified	SIRS + SBP	Patient Outcomes, Sepsis Treatment/Management
Nelson (2012)[125]	Conference Abstract	United States	single cohort	ED	4	24382	Age not specified	Unspecified	modified SIRS, SIRS + Organ dysfunction	Patient Outcomes, Sepsis Treatment/Management
Nguyen (2014)[126]	Journal Article	United States	single cohort	ED	1	1095	Age not specified	Homegrown	SIRS + Organ dysfunction	Patient Outcomes
Noonan (2018)[127]	Thesis	United States	single cohort	ED	1	6940	Age not specified	Unspecified	SIRS	Usability
Okafor (2014)[128]	Conference Abstract	Unspecified	single cohort	ED	2	3921	Age not specified	Unspecified	SIRS	Patient Outcomes
Oxman (2013)[129]	Conference Abstract	United States	before/after (not controlled)	Hospital-wide	NR	NR	Age not specified	Homegrown	Unspecified	Patient Outcomes, Sepsis Treatment/Management
Pandit (2015)[130]	Conference Abstract	Unspecified	Case control	Cancer center	1	806	Age not specified	Unspecified	Abnormal vital signs, SIRS	Patient Outcomes
Perlin (2020)[131]	Journal Article	United States	before/after (not controlled)	Inpatient wards (not ED)	173	NR	Age not specified	Homegrown	SIRS + sign of suspected infection	Patient Outcomes, Sepsis Treatment/Management
Pertiwi (2018)[132]	Journal Article	Unspecified	Heuristic evaluation	Hospital-wide	NR	NR	Age not specified	Commercial	SIRS + Organ dysfunction	Usability
Pham (2012)[133]	Conference Abstract	United States	Not enough information to determine	Specific ward	1	NR	Age not specified	Homegrown	Other + SIRS, SIRS	Patient Outcomes, Usability

Author (Year)	Type of publication	Country	Principal study type	Setting	Number of sites	Number of participants	Population	CCDS type	CCDS sepsis criteria	Outcome category
Powers (2014)[134]	Conference Abstract	Unspecified	single cohort	ICU, Inpatient wards (not ED)	NR	403	Age not specified	Homegrown	Other + SIRS	Patient Outcomes
Pulia (2016)[135]	Conference Abstract	Unspecified	before/after (not controlled)	ED	1	660	Adult	Unspecified	Abnormal vital signs	Sepsis Treatment/Management
Rauch (2013)[136]	Conference Abstract	Unspecified	single cohort	Surgical ICU	1	1119	Age not specified	Unspecified	modified SIRS	Patient Outcomes
Rincon (2017)[137]	Journal Article	United States	Focus groups	ICU	NR	NR	Age not specified	Homegrown	Other	Usability
Salamah (2013)[138]	Conference Abstract	Unspecified	single cohort	ED	1	8538	Age not specified	Homegrown	Other + SIRS	Patient Outcomes, Sepsis Treatment/Management
Sankey (2017)[139]	Conference Abstract	United States	before/after (not controlled)	Specific ward	1	15554	Adult	Unspecified	modified SIRS	Patient Outcomes
Santistevan (2017)[140]	Conference Abstract	United States	single cohort	ED	1	387	Adult	Homegrown	SIRS + Organ dysfunction + sign of suspected infection	Patient Outcomes
Sarumi (2013)[141]	Conference Abstract	United States	before/after (not controlled)	Inpatient wards (not ED or ICU)	1	NR	Age not specified	Unspecified	SIRS	Patient Outcomes
Sawyer (2011)[142]	Journal Article	United States	controlled study	Inpatient wards (not ICU)	6	300	Adult	Homegrown	Abnormal vital signs & laboratory results	Patient Outcomes, Sepsis Treatment/Management
Seetharaman (2019)[143]	Journal Article	United States	single cohort	Hospital-wide	5	10953	Adult	Unspecified	modified SIRS + organ dysfunction	Patient Outcomes, Sepsis Treatment/Management
Sharp (2016)_1[144]	Conference Abstract	Unspecified	before/after (not controlled)	ED	1	656	Adult	Unspecified	Nursing assesment + abnormal vital signs	Sepsis Treatment/Management
Sharp (2016)_2[145]	Conference Abstract	United States	single cohort	ED	1	1108	Adult	Unspecified	Other	Patient Outcomes
Sherwin (2014)[146]	Conference Abstract	Unspecified	single cohort	ED	2	16201	Age not specified	Unspecified	SIRS + Organ dysfunction	Patient Outcomes
Shetty (2021)[147]	Journal Article	Australia	single cohort	ED	5	118178	Adult	Homegrown	SIRS	Patient Outcomes
Song (2019)[148]	Journal Article	South Korea	before/after (not controlled)	ED	1	631	Adult	Homegrown	qSOFA + sign of suspected infection + SOFA	Patient Outcomes, Sepsis Treatment/Management
Swenson (2018)[149]	Conference Abstract	Unspecified	survey	Inpatient wards	1	121	Adult	Unspecified	Abnormal vital signs & laboratory results	Usability
Swenson (2019)[150]	Conference Abstract	United States	controlled study	Inpatient wards	1	2048	Age not specified	Unspecified	SIRS + Organ dysfunction + sign of suspected infection	Patient Outcomes, Sepsis Treatment/Management
Tafelski (2010)[151]	Journal Article	Germany	before/after (not controlled)	ICU	3	1158	Adult	Homegrown	Unspecified	Patient Outcomes, Sepsis Treatment/Management

Author (Year)	Type of publication	Country	Principal study type	Setting	Number of sites	Number of participants	Population	CCDS type	CCDS sepsis criteria	Outcome category
Thompson (2003)[152]	Journal Article	United States	controlled study	ICU	2	203	Age not specified	Homegrown	Other + SIRS, SIRS	Patient Outcomes, Sepsis Treatment/Management
Tsai (2015)[153]	Conference Abstract	Unspecified	single cohort	ED	1	13906	Age not specified	Homegrown	Unspecified	Patient Outcomes
Umscheid (2015)[154]	Journal Article	United States	before/after (not controlled)	Inpatient wards (not critical care)	3	31093	Adult	Homegrown	SIRS + Organ dysfunction	Patient Outcomes, Sepsis Treatment/Management
Watanabe (2017)[155]	Conference Abstract	United States	single cohort	Hospital-wide	1	100	Adult	Commercial	SIRS, SIRS + Organ dysfunction	Sepsis Treatment/Management
Wawrose (2016)[156]	Journal Article	United States	single cohort	Specific ward	1	348	Adult	Commercial	SIRS, SIRS + Organ dysfunction	Patient Outcomes
Westphal (2018)[157]	Journal Article	Brazil	before/after (not controlled)	Hospital-wide (not-ICU)	1	637	Age not specified	Homegrown	MEWS	Patient Outcomes, Sepsis Treatment/Management
Winterbottom (2016)[158]	Research Showcase abstract	Unspecified	Not enough information to determine	Inpatient wards	NR	NR	Age not specified	Unspecified	MEWS	Patient Outcomes, Sepsis Treatment/Management
Wolpaw (2018)[159]	Conference Abstract	United States	before/after (not controlled)	Acute care wards	NR	NR	Age not specified	Unspecified	Abnormal vital signs & laboratory results	Patient Outcomes, Sepsis Treatment/Management, Usability
Yeich (2012)[160]	Conference Abstract	United States	before/after (not controlled)	ED, Inpatient wards	1	NR	Age not specified	Unspecified	SIRS + Organ dysfunction, SIRS + shock	Patient Outcomes
Yohannes (2019)[161]	Conference Abstract	United States	before/after (not controlled)	Hospital-wide	1	4102	Age not specified	Unspecified	SIRS	Patient Outcomes, Sepsis Treatment/Management
Zaragoza (2019)[162]	Conference Abstract	Unspecified	single cohort	Hospital-wide	1	NR	Age not specified	Homegrown	Sepsis-2, Sepsis-3	Patient Outcomes
Zuick (2016)[163]	Journal Article	United States	single cohort	Inpatient wards (not ED or ICU)	1	97	Age not specified	Homegrown	Other	Patient Outcomes, Sepsis Treatment/Management

ICU=Intensive care unit, NR=not reported, SOFA=Sequential Organ Failure Assessment, ED=Emergency Department, MEWS=Modified Early Warning Score, SIRS= Systemic Inflammatory Response Syndrome, MAP=Mean Arterial Pressure, MEWS-SRS=Modified early warning signs-sepsis recognition score, RCT=Randomised Control Trial, SBP=Systolic blood pressure, NEWS=National Early Warning Score, MEDS=Mortality in emergency department sepsis score, REMS=Rapid emergency medicine score, SCS=Simple clinical score, qSOFA=Quick Sepsis-related Organ Failure Assessment.
