# Supplementary figures and images for: Computerized Clinical Decision Support Systems for the Early Detection of Sepsis Among Adult Inpatients: Scoping Review
Source: J Med Internet Res. 2022 Feb 23;24(2):e31083. doi: 10.2196/31083 (PMC8908200; doi:10.2196/31083)

## Multimedia appendix 6: Number of journal articles by country (n=65)

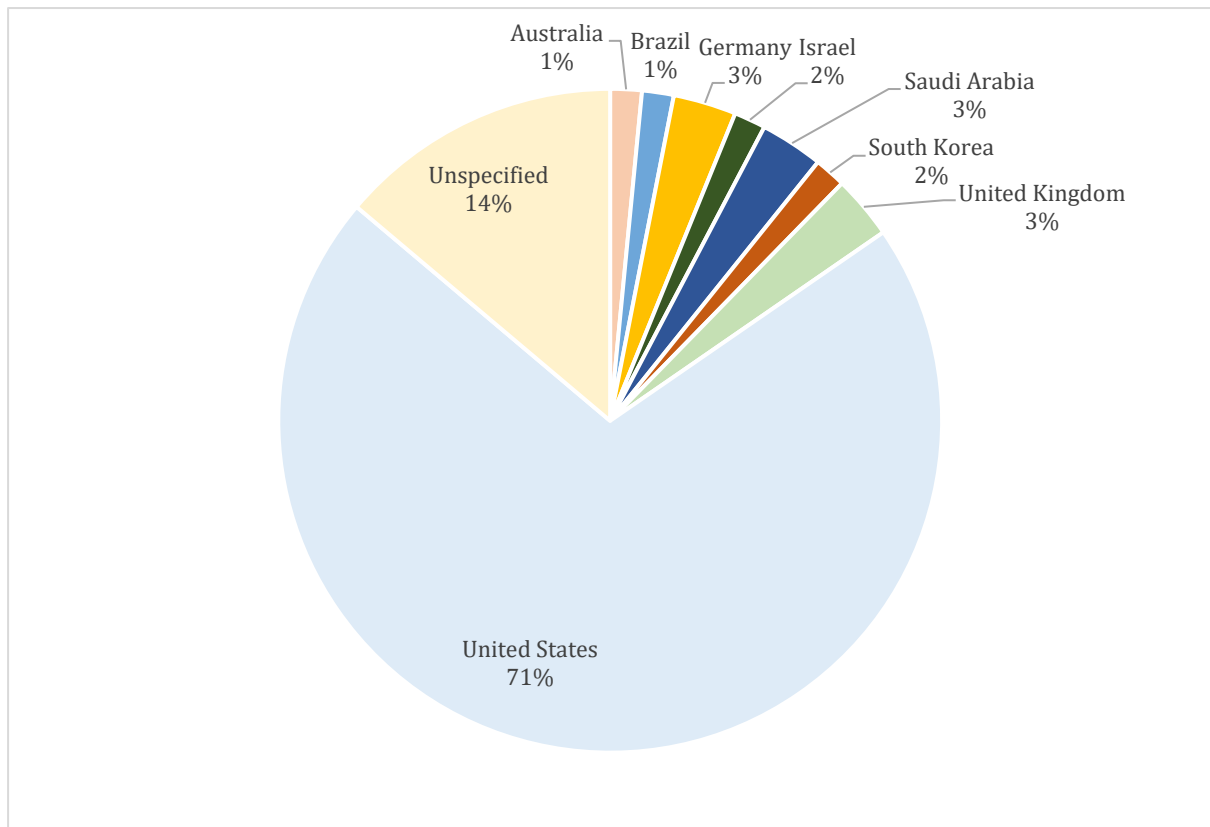

Supplement: Multimedia Appendix 6 [file jmir_v24i2e31083_app6.pdf]
