# Supplementary material for: Computerized Clinical Decision Support Systems for the Early Detection of Sepsis Among Adult Inpatients: Scoping Review
Source: J Med Internet Res. 2022 Feb 23;24(2):e31083. doi: 10.2196/31083 (PMC8908200; doi:10.2196/31083)
Supplement: Multimedia Appendix 7 [file jmir_v24i2e31083_app7.pdf]

Multimedia appendix 7: Main outcomes and outcome categories in gray literature

A) Conference abstracts (n=54)

Outcome category	Outcome classification[a], n(%[b])			Total, n (%[d])
	Primary	Secondary	Not specified[c]	
**Patient outcomes**				
Mortality	3 (13)	1 (4)	19 (83)	23 (43)
Sepsis identification	11 (42)	1 (4)	14 (54)	26 (48)
Length of stay	0 (0)	0 (0)	5 (100)	5 (9)
Intensive care unit admission	0 (0)	0 (0)	5 (100)	5 (9)
Other	1 (14)	0 (0)	6 (86)	7 (13)
**Sepsis Treatment and Management**				
Antibiotics	3 (27)	0 (0)	8 (73)	11 (20)
Lactate	0 (0)	0 (0)	1 (100)	1 (2)
Fluids	0 (0)	0 (0)	5 (100)	5 (9)
Blood culture	0 (0)	1 (33)	2 (67)	3 (6)
Sepsis bundle or protocol compliance	1 (13)	0 (0)	7 (88)	8 (15)
Other	1 (8)	1 (8)	10 (83)	12 (22)
**Usability**				
Efficiency	0 (0)	1 (50)	1 (50)	2 (4)
Effectiveness	0 (0)	1 (50)	1 (50)	2 (4)
Satisfaction	0 (0)	1 (25)	3 (75)	4 (7)
**Cost**				
Cost	0 (0)	0 (0)	1 (100)	1 (2)

B) Theses (n=5)

Outcome category	Outcome classification[a], n(%[b])			Total, n (%[d])
	Primary	Secondary	Not specified[c]	
**Patient outcomes**				
Mortality	0 (0)	1 (50)	1 (50)	2 (40)
Sepsis identification	0 (0)	0 (0)	3 (100)	3 (60)
Length of stay	0 (0)	1 (50)	1 (50)	2 (40)
Other	0 (0)	1 (50)	1 (50)	2 (40)
**Sepsis Treatment and Management**				
Antibiotics	0 (0)	0 (0)	2 (100)	2 (40)
Lactate	0 (0)	0 (0)	1 (100)	1 (20)
Fluids	0 (0)	0 (0)	1 (100)	1 (20)
Blood culture	0 (0)	0 (0)	2 (100)	2 (40)
Sepsis bundle or protocol compliance	0 (0)	0 (0)	1 (100)	1 (20)
Other	1 (33)	0 (0)	2 (67)	3 (60)
**Usability**				
Effectiveness	1 (100)	0 (0)	0 (0)	1 (20)

[a]Some studies reported both primary, secondary, or non-specified outcomes within the same outcome group. To avoid double-counting these studies, secondary outcomes were not counted in favor of counting primary outcomes. Similarly, non-specified outcomes were not counted in favor of primary or

secondary outcomes. For example, a study may have the primary outcome mortality (30-day) and the secondary outcome mortality (7-day), which would both fall into the mortality outcome group. In this example the study would be counted as having mortality as the primary outcome.

[b]These percentages were calculated as row percentages, i.e. using the total number in each row as the denominator.

[c]The study did not specify whether the outcomes were primary or secondary.

[d]The percentages were calculated from the number of conference abstracts (n=54) or theses (n=5), not the number of total outcomes. As many studies reported multiple outcomes the percentages will add up to more than 100%.
