# Supplementary material for: Computerized Clinical Decision Support Systems for the Early Detection of Sepsis Among Adult Inpatients: Scoping Review
Source: J Med Internet Res. 2022 Feb 23;24(2):e31083. doi: 10.2196/31083 (PMC8908200; doi:10.2196/31083)
Supplement: Multimedia Appendix 8 [file jmir_v24i2e31083_app8.pdf]

Multimedia appendix 8: Types of mortality reported in journal articles (n=65)

Mortality type	Times reported, n(%)
**Total**	**55**
Adjusted mortality	1 (2)
Hospital mortality rate	1 (2)
Mortality	6 (11)
Mortality (10-day) prediction	1 (2)
Mortality (1-day) prediction	1 (2)
Mortality (28-day in-hospital) prediction	1 (2)
Mortality (28-day)	1 (2)
Mortality (30-day all-cause)	2 (4)
Mortality (30-day in-hospital all-cause)	1 (2)
Mortality (30-day) prediction	1 (2)
Mortality (5-day) prediction	1 (2)
Mortality (60-day) prediction	1 (2)
Mortality (90-day)	1 (2)
Mortality (admitted in-patients with sepsis)	1 (2)
Mortality (all-cause 14 day)	1 (2)
Mortality (all-cause 7 day)	1 (2)
Mortality (emergency department admitted patients with sepsis)	1 (2)
Mortality (Health care facility-onset Clostridium difficile infections)	1 (2)
Mortality (hospital)	1 (2)
Mortality (intensive care unit admitted)	1 (2)
Mortality (intensive care unit)	1 (2)
Mortality (in-hospital 30-day)	1 (2)
Mortality (in-hospital post nurse practitioner rapid response team interaction)	1 (2)
Mortality (in-hospital)	13 (24)
Mortality (in-hospital) prediction	1 (2)
Mortality (inpatient)	1 (2)
Mortality (respiratory failure related)	1 (2)
Mortality (sepsis-related in-hospital)	1 (2)
Mortality (sepsis-related in-patient)	1 (2)
Mortality (sepsis-related)	3 (5)
Mortality (within 30 days of alert)	1 (2)
Mortality rate	1 (2)
Mortality within 48hr of alert	1 (2)
Risk-adjusted mortality index	1 (2)
Unadjusted mortality	1 (2)
